# Adsorptive behavior of poly (vinylidene fluoride) membranes for the recovery of lignin-derived hydrophobic deep eutectic solvents

**DOI:** 10.1038/s41598-025-18164-x

**Published:** 2025-09-01

**Authors:** Odianosen I. Ewah, Yuxuan Zhang, Jian Shi, Isabel C. Escobar

**Affiliations:** 1 Department of Chemical and Materials Engineering, University of Kentucky, Lexington, KY 40506 USA; 2 Biosystems and Agricultural Engineering, University of Kentucky, 128 C.E. Barnhart Building, Lexington, KY 40506 USA

**Keywords:** Hydrophobic deep eutectic solvents, Polyvinylidene fluoride, Hansen solubility parameters, Membrane separations, Lignin separation, Adsorption kinetics, Solvent-resistant membranes, Engineering, Materials science

## Abstract

Recently, membrane technology has gained significant traction as an energy-efficient alternative to traditional thermal processes for solvent recovery. Deep eutectic solvents (DESs) have emerged as sustainable alternatives to conventional organic solvents, yet a systematic methodology for selecting compatible membrane materials for their recovery remains underdeveloped. This study established a predictive framework for membrane material selection in hydrophobic DES applications using Hansen Solubility Parameters (HSP) with inverted criteria targeting materials with relative energy difference (RED) values greater than 1.0. Flat sheet membranes were fabricated via the non-solvent induced phase separation (NIPS) technique. Four NIPS fabricated polymer membranes were evaluated: polysulfone, cellulose acetate, polyvinylidene fluoride (PVDF) fabricated with polyethylene glycol (PEG) as a pore-forming agent, and polybenzimidazole (PBI). The HSP approach successfully predicted membrane-solvent compatibility, with polysulfone (RED = 0.6) and cellulose acetate (RED = 0.9) dissolving completely within 24 h, while PVDF (RED = 1.9) and PBI (RED = 1.1) maintained structural integrity throughout a 7-day exposure period. Furthermore, PVDF demonstrated superior performance with minimal weight gain (3.0%), hydrophobic surface characteristics (122° water contact angle), and enhanced mechanical properties following DES exposure. Comprehensive chemical and morphological characterization confirmed PVDF’s chemical stability and revealed a surface-selective interaction mechanism involving simultaneous PEG (pore-forming agent) extraction and DES component adsorption. Adsorption kinetics followed pseudo-first-order behavior with reversible characteristics, best described by the Temkin isotherm model (R² = 0.9987). PVDF membranes-maintained separation functionality with average lignin rejection (75.2 ± 7.69%) and demonstrated filtration permeability of 2.0 ± 0.34 LMH/bar. This methodology provides a rational approach for membrane selection in emerging solvent systems, contributing to the advancement of sustainable separation technologies for DES-based biomass processing applications.

**Supplementary Information:**

The online version contains supplementary material available at 10.1038/s41598-025-18164-x.

## Introduction

Deep eutectic solvents (DESs) have emerged as a significant breakthrough in green chemistry over the past two decades. These solvents, formed through the complexation of hydrogen bond acceptors (HBAs) with hydrogen bond donors (HBDs), exhibit a substantial melting point depression compared to their individual components^1^. This depression allows the mixture to remain liquid at room temperature, enabling their use as solvents without the need for heating or additional processing. DESs are increasingly recognized as sustainable alternatives to conventional volatile organic solvents, offering advantages including low volatility, biodegradability, non-flammability, and tunable physicochemical properties^2^. Their versatility has expanded their application across various fields, including catalysis, extraction, electrochemistry, and more recently, membrane technology^3,4^.

DESs are typically classified based on their composition, with the most common categories including Type I (metal salt + organic salt), Type II (metal salt hydrate + organic salt), Type III (organic salt + HBD), and Type IV (metal salt hydrate + HBD)^5^. Of particular interest are hydrophobic DESs (HDESs), a relatively recent subclass that addresses a significant limitation of conventional hydrophilic DESs—their instability upon contact with water^6^. HDESs represent a promising solution for applications involving water-immiscible processes, particularly in extraction and separation technologies^7^. Recent innovations have focused on developing HDESs from renewable sources, such as lignin derivatives, further enhancing their sustainability profile while capitalizing on abundant biowaste streams^8,9^.

Despite the growing interest in DES applications, a critical methodological gap exists in the selection of compatible membrane materials for specific DES systems, particularly for emerging solvents like hydrophobic DESs^10^. Membrane technology offers an energy-efficient approach to DES processing and recovery compared to traditional thermal separation methods, making it a promising solution for industrial applications^11^. However, the successful implementation of membrane technology for DES systems hinges on selecting membrane materials that maintain their structural integrity and functionality when exposed to these solvents^12^.

The selection of solvent-resistant membrane materials requires careful consideration of their chemical stability, mechanical properties, and separation performance in specific solvent environments. Several polymer materials have demonstrated promising capabilities in this regard. Polybenzimidazole (PBI) membranes, extensively studied by Livingston and colleagues, exhibit exceptional chemical stability in harsh organic solvents when properly crosslinked with dibromoxylene or other crosslinking agents^13,14^. These membranes retain their separation performance even in aggressive solvents like dimethylformamide (DMF) and N-methyl-2-pyrrolidone (NMP)^15^. Cellulose acetate, one of the earliest materials used for organic solvent filtration, offers good processability but requires modification through crosslinking to enhance its resistance to chemicals and mechanical strength^16^. Polysulfone-based membranes provide excellent mechanical stability and can be made solvent-resistant through UV-curing techniques that create semi-interpenetrating networks^17^. Polyvinylidene fluoride (PVDF) has emerged as a particularly promising candidate due to its inherent chemical resistance, thermal stability, and compatibility with various crosslinking methods that further enhance its solvent resistance^18,19^. Despite these advances in solvent-resistant membrane materials, a systematic methodology for selecting the optimal membrane material for a specific solvent system remains largely underdeveloped, highlighting the need for more predictive approaches that can guide material selection beyond empirical testing.

The compatibility between membrane materials and solvents is governed by complex interactions, including chemical resistance, physical stability, and potential adsorption phenomena^20^. Conventional methods for predicting polymer-solvent compatibility often rely on thermodynamic principles such as Hansen Solubility Parameters (HSP) and the Relative Energy Difference (RED) approach^21,22^. The RED methodology provides a quantitative assessment of polymer-solvent interactions through the dispersive, polar, and hydrogen bonding components of the solubility parameters^21^. In conventional applications of this approach, when RED < 1, the solvent is expected to dissolve or significantly swell the polymer, indicating incompatibility for membrane applications^23^. Conversely, the deliberate ‘flip’ of targeting materials with RED values greater than 1 enables the identification of membranes that will maintain their structural integrity when exposed to solvents while still allowing for controlled interactions at the interface. This inverted approach could be validated through comprehensive experimental characterization of membrane-solvent interactions, specifically focusing on polymeric membranes that have demonstrated promising solvent resistance properties in literature.

The selection of PVDF, polysulfone, cellulose acetate, and PBI for screening in this study was guided by several critical factors. First, their historical performance in membrane technology applications demonstrates proven functionality and reliability^24^. Second, their documented chemical and solvent resistance properties suggest potential compatibility with emerging solvents like hydrophobic DESs^25^. Third, their mechanical stability under typical processing conditions ensures operational durability^26^. Fourth, their commercial availability and processability facilitate potential scale-up and practical implementation^27^. Lastly, their previous success in similar solvent systems provides a foundation for extension to hydrophobic DES applications^28^. However, while these polymers show potential for chemical compatibility with other solvents, their interaction with DES will extends beyond simple resistance to degradation.

In the context of membrane-based separations involving DESs, understanding possible adsorption phenomena is particularly crucial. Adsorption of DES components onto the membrane surface or within its porous structure can impact membrane flux, rejection and overall separation efficiency^29^. For hydrophobic DESs derived from lignin, which contain complex aromatic structures with various functional groups, adsorption interactions with membrane materials may be particularly pronounced and require systematic investigation.

The present study aims to address these knowledge gaps by establishing a methodological approach for membrane material selection for hydrophobic DES systems, with a specific focus on validating PVDF compatibility. The research objectives include: (1) developing and applying a systematic screening methodology based on RED calculations to identify candidate membrane materials for lignin-derived hydrophobic DESs; (2) experimentally validating the chemical compatibility of selected materials through comprehensive characterization of their physical, chemical, thermal and surface properties before and after DES exposure; and (3) investigating the adsorptive behavior of PVDF membranes with the hydrophobic DES. This multi-faceted approach provides valuable insights for the rational design of membrane processes for hydrophobic DES applications, contributing to the advancement of sustainable separation technologies.

## Experimental section

### Materials

Polysulfone (PSf, average Mw 35,000 by light scattering, average Mn 16,000 by membrane osmometry, pellets), cellulose acetate, kraft lignin, thymol (Thy) (≥ 98%), 2,6-dimethoxyphenol (Dmp) (≥ 98%), n-hexane, ethyl alcohol and poly (ethylene glycols) (PEGs, MW 1000 g/mol) were purchased from Sigma-Aldrich (Saint Louis, MO, USA). N-methyl-2-pyrrolidone (NMP), N, N-dimethylacetamide (DMAc) and N, N-dimethylformamide (DMF) were obtained from VWR. Polyvinylidene fluoride (PVDF/KYNAR) was obtained from Arkema Incorporation (Radnor, PA, USA). A solution containing 26 wt% polybenzimidazole (PBI, Celazole S26) (MW 27,000 g/mol) and 1.5 wt% lithium chloride (LiCl) in N, N-dimethylacetamide (DMAc) was purchased from PBI Performance Products Inc.

### Thermodynamics

#### Hansen solubility parameter (HSP) calculation

To identify suitable solvents for a specific polymer, the polymer must demonstrate adequate solubility or dispersion characteristics in the target solvent^21^. For assessing potential solvent-polymer compatibility, the relative energy difference (RED) is determined using Eq. (1):

1 $$\:RED\:=\:\frac{{R}_{a}\:}{R}_{0}$$

where $\:{R}_{0}$ represents the interaction radius of a Hansen solubility parameter sphere and $\:{R}_{a}\:$ denotes the solubility parameter distance between polymer (1) and solvent (2). The parameter Ra is computed from the individual Hansen solubility parameters ($\:{\delta\:}_{d}\:$for dispersive forces, $\:{\delta\:}_{p\:}$for polar forces, and $\:{\delta\:}_{h\:}$ for hydrogen bonding interactions) using Eq. (2)^21^:

2 $$\:{R}_{a}=\sqrt{4({{\delta\:}_{d2}-{\delta\:}_{d1})}^{2}+({{\delta\:}_{p2}-{\delta\:}_{p1})}^{2}+({{\delta\:}_{h2}-{\delta\:}_{h1})}^{2}}$$

**Fig. 1:**
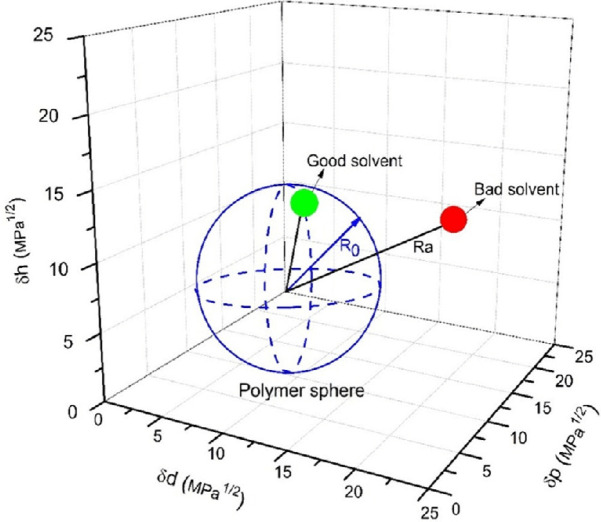
The Hansen solubility sphere space illustrating polymer-solvent compatibility for membrane fabrication applications with good and poor solvent classification^30^.

Enhanced solubility is indicated as Ra approaches zero^21^. The Hansen Solubility Parameter methodology represents polymer-solvent interactions within a three-dimensional coordinate system where each material is characterized by its dispersive ($\:{\delta\:}_{d}$), polar ($\:{\delta\:}_{p\:}$), and hydrogen bonding ($\:{\delta\:}_{h\:}$) components, as demonstrated in Fig. 1^30^. In membrane fabrication, solvents positioned inside the polymer’s solubility sphere are classified as effective solvents that promote dissolution or significant swelling, whereas those external to the sphere are categorized as poor solvents or non-solvents.

The calculation of Hansen solubility parameters for DESs requires consideration of the individual HSP contributions from each component based on their molar fractions in the mixture. Following the methodology established in literature^21,31^, the HSP parameters for a binary DES system can be calculated using the weighted average approach shown in Eqs. (3–5):

3 $$\:{\delta\:}_{d}=\:{n}_{i}{\delta\:}_{di}+\:{n}_{j}{\delta\:}_{dj}$$

4 $$\:{\delta\:}_{p}=\:{n}_{i}{\delta\:}_{pi}+\:{n}_{j}{\delta\:}_{pj}$$

5 $$\:{\delta\:}_{h}=\:{n}_{i}{\delta\:}_{hi}+\:{n}_{j}{\delta\:}_{hj}$$

where n represents the molar fraction of each DES component, with subscript i denoting component 1 (thymol in this case) and subscript j representing component 2 (2,6-dimethoxyphenol). The molar fractions are derived from the molar ratio used to prepare the DES, ensuring that ni + nj = 1. This additive approach assumes that the Hansen solubility parameters of the eutectic mixture can be approximated as the weighted sum of the individual component parameters, with each component contributing proportionally to its molar fraction in the mixture. The resulting calculated HSP values (δd, δp, δh) for the DES can then be used in Eq. (2) to determine the solubility parameter distance (Ra) between the DES and candidate membrane polymers, enabling the prediction of polymer-solvent compatibility through the RED methodology.

**Fig. 2:**
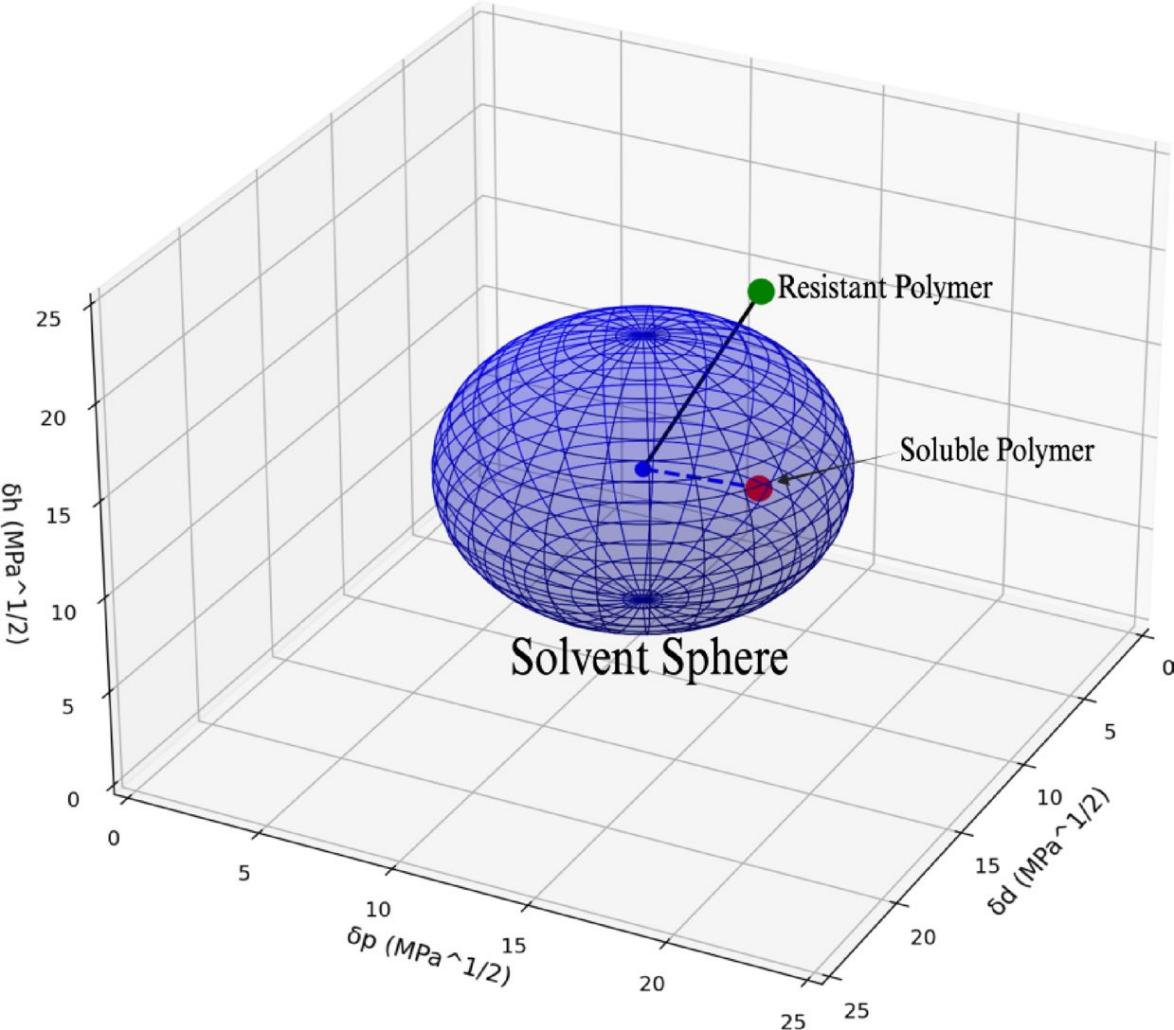
The Hansen solubility sphere space demonstrating the inverted solvent resistance membrane approach for identifying chemically stable polymers.

For membrane applications requiring solvent resistance, it is proposed here that this traditional interpretation can be strategically reversed. As illustrated in Fig. 2, polymers located outside the solvent sphere are identified as resistant materials capable of maintaining structural integrity upon solvent exposure, while those within the sphere would experience dissolution. This inverted approach enables the rational selection of solvent-resistant polymeric membranes by targeting materials with RED values greater than 1, to predict membrane stability in challenging solvent environments.

### Preparation of deep eutectic solvent (DES)

Thymol and 2,6-dimethoxyphenol (Dmp) were combined at a 1:1 molar ratio through careful weighing and premixing. The binary components were sealed within a glass vessel, and the mixture underwent heating at 80 °C for 1 h using an oil bath. Upon achieving a homogeneous liquid state, the resulting solution was cooled to ambient temperature, following established protocols described by Zhang et al.^9^. The physicochemical properties of the prepared DES have been comprehensively characterized, showing density of 1.077 ± 0.002 g/mL at ambient temperature, viscosity of 34.91 ± 0.26 mPa s at 25 °C, glass transition temperature of − 58.2 °C, and hydrophobic nature with minimal water solubility (0.80 ± 0.20 wt% leaching into aqueous phase).

### Preparation of flat sheet membranes

Flat sheet membranes were fabricated via the non-solvent induced phase separation (NIPS) technique. For polysulfone (PSf) and cellulose acetate membranes, predetermined masses of the respective polymers were dissolved in NMP and DMF solvents to create uniform dope solutions. For polyvinylidene fluoride (PVDF) membranes, polyethylene glycol 2000 (PEG2000) was incorporated as a pore-forming agent into the dope solution, utilizing a 50:50 volumetric mixture of DMAc and DMF as co-solvents to enhance polymer solubilization and membrane morphology. The polybenzimidazole (PBI) dope solution, initially supplied at 26 wt% from PBI Performance Products Inc., underwent dilution to reduce viscosity and facilitate casting operations. Following the achievement of homogeneous dope solutions, the mixtures were cast onto glass substrates using a doctor blade, immediately followed by immersion in a deionized water coagulation bath to initiate phase separation and membrane formation. The formed membranes were subsequently stored in deionized water for two nights, with daily water replacement to facilitate complete removal of residual solvents from the membrane matrix, before being used.

### Membrane screening and selection

#### Solubility and weight change in deep eutectic solvent (DES)

The four membranes samples (PSf, cellulose acetate, PVDF, and PBI) underwent immersion testing in the prepared deep eutectic solvent to assess their chemical stability and compatibility. Membrane samples were oven dried at 35 °C overnight, then weighed before submerged in the solvent solutions for a 7-day period, during which their dissolution behavior was monitored through visual observation and weight change analysis for membranes that demonstrated visual resistance to immediate dissolution. The membranes that did not dissolved immediately were taken out periodically at 24 h, 48 h, 72 h and 1 week and where washed three times with DI water and oven dried at 35 °C overnight before reweighed.

#### Wettability

Surface wettability characteristics of the fabricated membranes were determined through contact angle measurements utilizing a Kruss drop-shape analyzer DSA1005 (Matthews, NC, USA). The sessile drop technique was employed with a standardized 1 µL droplet volume per membrane sample, with contact angle measurements recorded immediately upon droplet deposition and at 10-second intervals thereafter. Prior to analysis, membrane samples underwent thorough rinsing with deionized water followed by overnight drying at ambient temperature. All measurements were conducted in triplicate to ensure reproducibility.

### Characterization of membrane and DES interactions

#### Fourier transform infrared (FTIR) spectroscopy

Chemical structural analysis of the prepared membranes was conducted using a Thermo Scientific Nicolet iS50 Fourier transform infrared (FTIR) spectrometer (Thermo Scientific, Waltham, Massachusetts, USA). Spectral data were collected through attenuated total reflectance mode, acquiring 64 scans per sample to ensure adequate signal-to-noise ratio. Prior to analysis, membrane samples underwent 24-hour air-drying to eliminate moisture interference.

#### Tensile strength testing

Mechanical integrity assessment of membranes before and after DES exposure was performed to determine the maximum tensile force withstand-able prior to failure. Mechanical characterization was conducted using an Instron tensile testing machine 2716-010 (Norwood MA, USA) configured with a maximum load capacity of 5 N and operational temperature range spanning − 70  to 250 °C^32^.

#### Thermogravimetric analysis (TGA)

Thermal stability evaluation of membranes pre- and post-DES exposure was accomplished through thermogravimetric analysis using a TA Instruments TGA 550 (New Castle, DE, USA). Experimental conditions maintained a nitrogen atmosphere with gas flow rates between 10 and 20 mL/min to prevent oxidative degradation during analysis.

#### Differential scanning calorimetry (DSC)

Thermal property characterization of membranes was performed using DSC (TA instruments, DSC 250, New Castle, DE, USA) to evaluate thermal transitions before and after solvent exposure. Dried membrane samples were analyzed in sealed platinum pans across a temperature range of − 50 to 200 °C, employing a controlled heating rate of 5 °C/min.

#### Scanning electron microscopy (SEM)

Morphological investigation of membrane structure was conducted using SEM Quanta FEG 250, FEI/ThermoFisher Scientific (Hillsboro, OR, USA). Cross-sectional samples were prepared via cryofracture techniques and subjected to platinum coating under vacuum conditions prior to imaging to ensure optimal conductivity and image quality.

#### X-ray photoelectron spectroscopy (XPS)

Surface chemical composition analysis of membrane top layers following DES exposure was performed using Thermo-Scientific K-Alpha X-ray photoelectron spectroscopy (Waltham, MA, USA). Depth profiling analysis incorporating ion beam trek etching was additionally conducted to investigate potential DES deposition patterns within the membrane structure.

### Adsorption experiments

#### Kinetic adsorption models

For adsorption studies, the DES was dissolved in n-hexane solvent as a carrier medium due to its ability to dissolve the DES components and enable accurate concentration measurements using spectrophotometric analysis^9,33^. Given that the DES components contained lignin aromatic compounds, quantification was achieved through ultraviolet-visible spectroscopy monitoring at a secondary wavelength of approximately 326 nm, consistent with established literature protocols for lignin quantification^34^. Adsorption kinetic investigations were conducted by systematically varying temperature (15, 25, and 40 °C) and initial concentration (20 v/v%, 50 v/v%, and 80 v/v%) as detailed in Table 1. Residual DES concentrations were monitored at predetermined time intervals.

**Table 1:** Adsorption experimental conditions.

Temperature (°C)	DES concentration (v/v%)
15	20
Room temperature (25)	50
40	80

The fundamental kinetic behavior of adsorption phenomena can be described through Langmuir kinetic theory, which characterizes adsorption rates based on the premise that molecular adsorption occurs at discrete binding sites on the adsorbent surface^35^. The Langmuir kinetic framework incorporates both adsorption and desorption processes as well as the pseudo first-order and pseudo second-order models in establishing a mechanistic foundation for understanding surface interaction dynamics^35,36^.

For quantitative analysis of adsorption kinetics, experimental time-series data are commonly evaluated using pseudo first-order and pseudo second-order kinetic models^37^. The pseudo first-order model yields information regarding both kinetic behavior and equilibrium conditions, operating under the assumption that adsorption rate demonstrates direct proportionality to time, with primary applicability during initial adsorption phases^37,38^. The mathematical expression for the pseudo first-order model is presented in Eq. (6):

6 $$q{\text{}} = {\text{}}q_{m} \left( {1{\text{}} - {\text{}}e^{{\left( { - K_{1} t} \right)}} } \right)$$

where $\:{q}_{m}$ represents the maximum adsorption capacity in µg/g, K_1_ denotes the rate constant in min⁻¹, and t signifies time in minutes.

The pseudo second-order kinetic model operates under the assumption that adsorption rate depends upon adsorption capacity rather than solution concentration, identifying chemical adsorption as the rate-determining step^39,40^. The pseudo second-order model is mathematically represented by Eq. (7):

7 $$q{\text{}} = {\text{}}\frac{{\left( {q_{m}^{2} K_{2} t} \right)}}{{\left( {1{\text{}} + {\text{}}q_{m} K_{2} t} \right)}}{\text{}}$$

where $\:{q}_{m}$ indicates the maximum adsorption capacity in µg/g, K_2_ represents the rate constant in g/(µg hr), and t denotes time in minutes.

#### Adsorption experiments for isotherms

Equilibrium adsorption experiments were conducted to establish the optimal adsorption mechanism and determine maximum adsorption capacity of membranes. Based on kinetic study outcomes, DES solutions were prepared in n-hexane at concentration ranges spanning 70 v/v% to 90 v/v%, as enhanced adsorption behavior was observed at elevated concentrations during kinetic evaluations. Solution mixtures were maintained in an Innova 4000 incubator shaker (Edison, NJ, USA) at 15 °C and 60 rpm for 8-hour equilibration periods, representing conditions that demonstrated optimal adsorption from kinetic data. All experimental procedures were performed in triplicate to ensure statistical validity. Concentration analysis was performed using a VWR^®^ UV-6300 PC double-beam spectrometer (Radnor, PA, USA), with adsorption capacity calculations following Eq. (8)^41,42^:

8 $$q{\text{}} = {\text{}}\frac{{C_{0} {\text{}} - {\text{}}Ce}}{m} \times {\text{}}V$$

where $\:q$ represents adsorption capacity in µg/g, $\:C_0$ indicates initial DES concentration in ppm, $\:Ce$ denotes final DES concentration in ppm, $\:V$ represents solution volume, and $\:m$ signifies membrane mass.

Isotherm modeling was accomplished using Langmuir, Freundlich, and Temkin mathematical models. The Langmuir isotherm^43^ (Eq. 9) describes equilibrium relationships between adsorbate and substrate, assuming monolayer adsorption limitations. This model incorporates assumptions of negligible lateral interactions between adsorbed molecules, monolayer coverage, homogeneous active site distribution, and uniform adsorption energy:

9 $$\:q\:=\:\frac{{q}_{m}Ce}{{K}_{d}\:+\:Ce}$$

where q represents adsorption capacity in µg/g, $\:{q}_{m}$ indicates maximum adsorption capacity in µg/g, $\:Ce$ denotes final DES concentration in mg/L, and $\:{K}_{d}$ represents the Langmuir adsorption equilibrium constant in L/g.

The Freundlich isotherm model^44,45^ (Eq. 10) accommodates surface heterogeneity effects, potentially arising from multilayer adsorption phenomena or exponential distribution of adsorbent active sites:

10 $$\:q\:=\:K{Ce}^{(1/n)}$$

where $\:q$ represents adsorption capacity in µg/g, $\:K$ indicates the Freundlich constant in L/mg, $\:Ce$ denotes final DES concentration in mg/L, and n represents the heterogeneity factor.

Lastly, the Temkin isotherm model^46^ (Eq. 11) incorporates temperature effects on adsorption processes and assumes linear decrease in adsorption heat with increasing surface coverage:

11 $$\:q\:=\:A\:+\:BlnC$$

where $\:q$ represents adsorption capacity in µg/g, $\:A$ and $\:B$ are Temkin constants, and $\:C$ denotes the equilibrium concentration in mg/L.

### Performance analysis: permeability and rejection

Water flux experiments were conducted at a constant pressure of 6 bar in an Solvent-resistant dead-end glass filtration cell (Solvent-resistant Stirred Cell 47 mm, Millipore Sigma Company, Burlington, MA, USA) to evaluate adsorption effects at each exposure interval. Membrane preconditioning procedures involved filtering 20 mL DES at a constant pressure of 6 bar for operational consistency. Following preconditioning, solutions containing 100 ppm kraft lignin dissolved in DES were filtered through membrane samples at a constant pressure of 6 bar and 50 °C to reduce DES viscosity for enhanced permeability characteristics. Permeability measurements were recorded at 5-mL filtration intervals, with permeate sample collection for lignin concentration analysis in both feed and permeate streams using UV-VIS spectroscopy (UV-6300PC, Leuven, Belgium). Lignin rejection efficiency was calculated using Eq. (12)^47^:

12 $$\:R\left(\text\%\right)\:=\:\frac{{C}_{f}\:-\:{C}_{p}}{{C}_{f}}\:\times 100$$

where $\:R\left(\%\right)\:$ represents rejection percentage, $\:{C}_{f}\:$indicates feed concentration in ppm, and $\:\:{C}_{p}$ denotes permeate concentration in ppm. All experimental procedures were conducted in triplicate for statistical reliability.

## Results and discussion

### Hansen solubility parameters calculations

The Hansen solubility parameters (HSP) for the DES and its individual components were calculated using the weighted average approach based on their molar fractions using Eqs. (3–5), as shown in Tables 2 and 3, reflecting the intermediate solubility characteristics of the binary eutectic mixture. It is noteworthy that the HSP approach operates under several assumptions, including ideal mixing behavior and additive contributions of individual components, which may not accurately reflect complex molecular interactions in eutectic systems^22^. Additionally, the HSP methodology assumes spherical solubility domains and uniform interaction energies, which can introduce uncertainties in predicting polymer-solvent compatibility for complex solvent systems^21,48^.

The relative energy difference (RED) calculations revealed significant variations in polymer-DES compatibility (Table 3). Polysulfone and cellulose acetate exhibited RED values of 0.6 and 0.9, respectively, both falling below the critical threshold of 1.0, indicating high affinity between these polymers and the DES. RED values less than 1.0 indicate that the solvent would dissolve the polymer, with RED numbers approaching zero representing greater potential for polymer dissolution^48^. This polymer-solvent compatibility renders polysulfone and cellulose acetate unsuitable for membrane applications requiring solvent resistance. In contrast, PVDF and PBI demonstrated RED values of 1.9 and 1.1, respectively, both exceeding the threshold value of 1.0, which is the focus of this study for identifying DES-resistant membrane materials. The inverted Hansen solubility parameter approach suggested PVDF and PBI as promising membrane materials for hydrophobic DES applications, with their RED values greater than 1 indicating that the polymers lie well outside the DES solubility sphere, predicting minimal swelling and dissolution effects.

**Table 2:** Hydrophobic deep eutectic solvent and its components Hansen solubility parameters.

DES Components	δd (MPa^0.5^)	δp (MPa^0.5^)	δh (MPa^0.5^)
Thymol^21^	19.0	4.5	10.8
2,6-dimethoxyphenol^21^	19.3	7.6	13.7
Deep eutectic solvent (Thy: Dmp)	19.2	6.1	12.3

**Table 3:** Polymers Hansen solubility parameters and R.E.D values.

Polymer	δd (MPa^0.5^)	δp (MPa^0.5^)	δh (MPa^0.5^)	*R*₀	Ra	*R*.E. D
Polysulfone^21^	19.7	8.3	8.3	8	4.7	0.6
Cellulose acetate^21^	18.2	12.4	10.8	7.4	6.8	0.9
PVDF^21^	17.0	12.1	10.2	4.1	7.7	1.9
PBI^49^	17.3	8.7	8.9	5.0	5.7	1.1

### Membrane screening and selection results

**Fig. 3:**
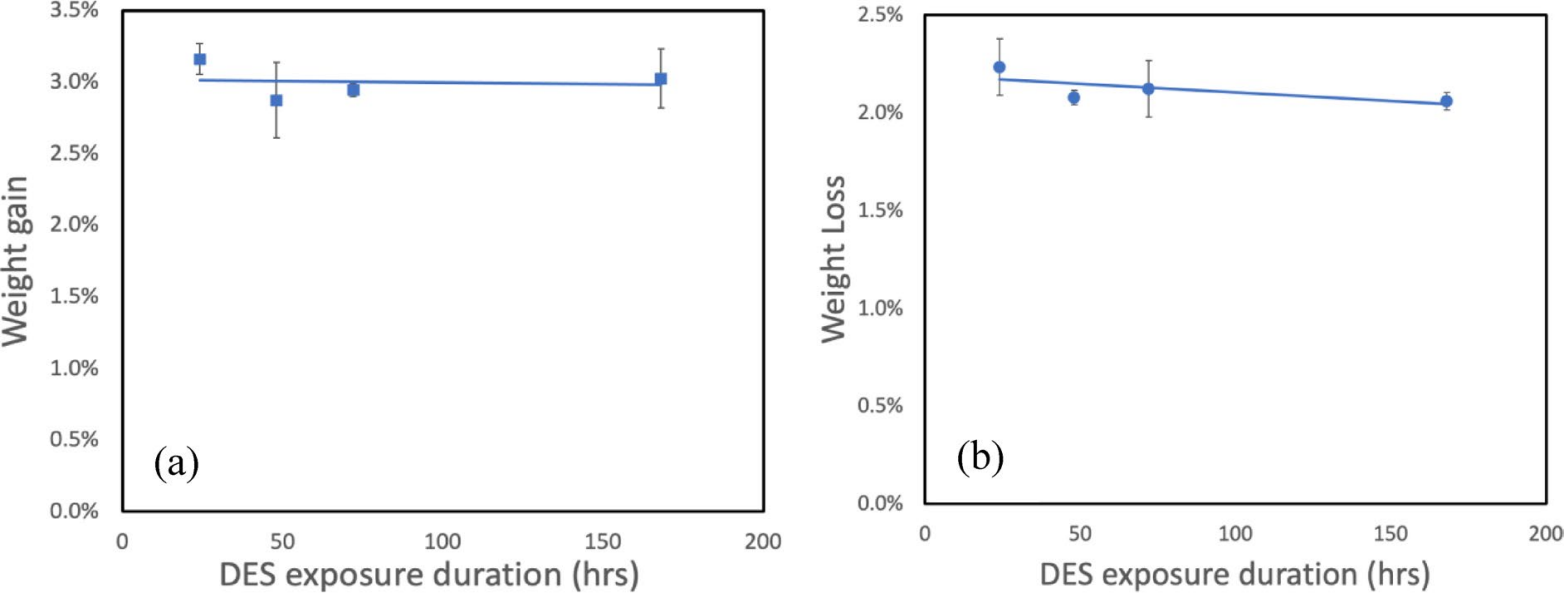
Weight change profiles during DES exposure: (**a**) PVDF membrane demonstrating weight gain behavior and (**b**) PBI membrane showing weight loss trends.

Based on the Hansen solubility parameter calculations in “Hansen solubility parameters calculations” section, all membrane polymers with calculated RED values were exposed to the DES for solubility testing over a 7-day duration. After 24 h, cellulose acetate and polysulfone completely dissolved in the DES, which aligned with their RED values of 0.9 and 0.6, respectively, confirming the predictive capability of the Hansen solubility parameter approach. In contrast, PVDF and PBI remained visually intact without dissolution in the DES, as shown in supplementary Figures S1, consistent with their RED values greater than 1.0. To further evaluate the chemical stability of the non-dissolved membranes, weight change analysis was conducted, as established by literature^50^. The weight change profiles for PVDF and PBI membranes are presented in Fig. 3a and b, respectively. PVDF membranes exhibited a weight gain of approximately 3.2% after initial exposure, which stabilized at approximately 3.0% throughout the remaining exposure duration (Fig. 3a). This minimal weight gain indicates limited solvent sorption, suggesting good chemical compatibility^51^. Conversely, PBI membranes demonstrated a weight loss of approximately 2.1% during DES exposure (Fig. 3b), which may be attributed to the leaching or dissolution of residual processing aids or minor structural rearrangements rather than chemical degradation^52^.

**Fig. 4:**
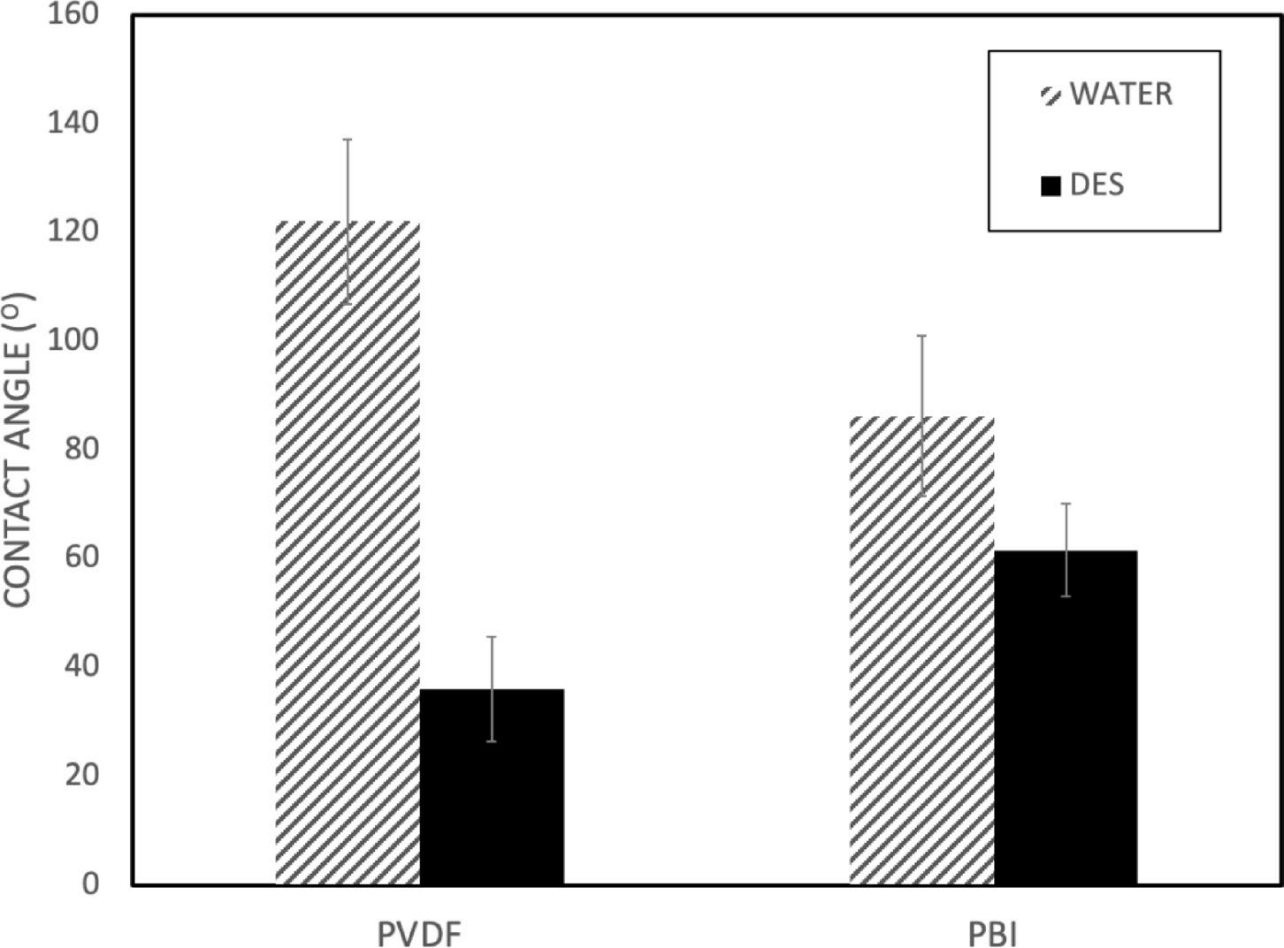
Contact angle measurements of PVDF and PBI membranes with water and DES demonstrating surface wettability characteristics.

To select membranes capable of filtering the hydrophobic DES, contact angle measurements were performed for both PVDF and PBI membranes to assess their surface wettability characteristics. The contact angle results are presented in Fig. 4, showing that PVDF membranes exhibited a water contact angle of 122°, indicating a hydrophobic surface^53^, that is favorable for hydrophobic applications. PBI membranes demonstrated lower hydrophobicity with contact angles of 86° for water^54^ and 62° for DES, suggesting moderate wettability. The lower contact angles observed with DES for both membranes indicates preferential affinity toward the hydrophobic solvent system, which is advantageous for separation applications involving hydrophobic DES.

However, with the focus of this study being polymeric membrane resistance to this DES, PVDF was selected for further investigation due to its superior chemical stability as demonstrated by the weight analysis, which can be attributed to its strong and chemically inert C-F bonds. It is noteworthy that this study demonstrated short-term compatibility over a 7-day exposure period following established literature protocols^55–57^, though long-term exposure periods remain an important area for future investigation. Additionally, PVDF exhibited a lower membrane-DES contact angle indicating enhanced wettability by the DES^58^ as compared to PBI that demonstrated weight loss and a higher membrane-DES contact angle, indicating lower DES wettability. Furthermore, PVDF membranes possess established commercial scalability, representing two-thirds of the current membrane filtration market^18^, in contrast to the more specialized PBI membranes^59^.

### Characterization of PVDF membrane and DES interaction

**Fig. 5:**
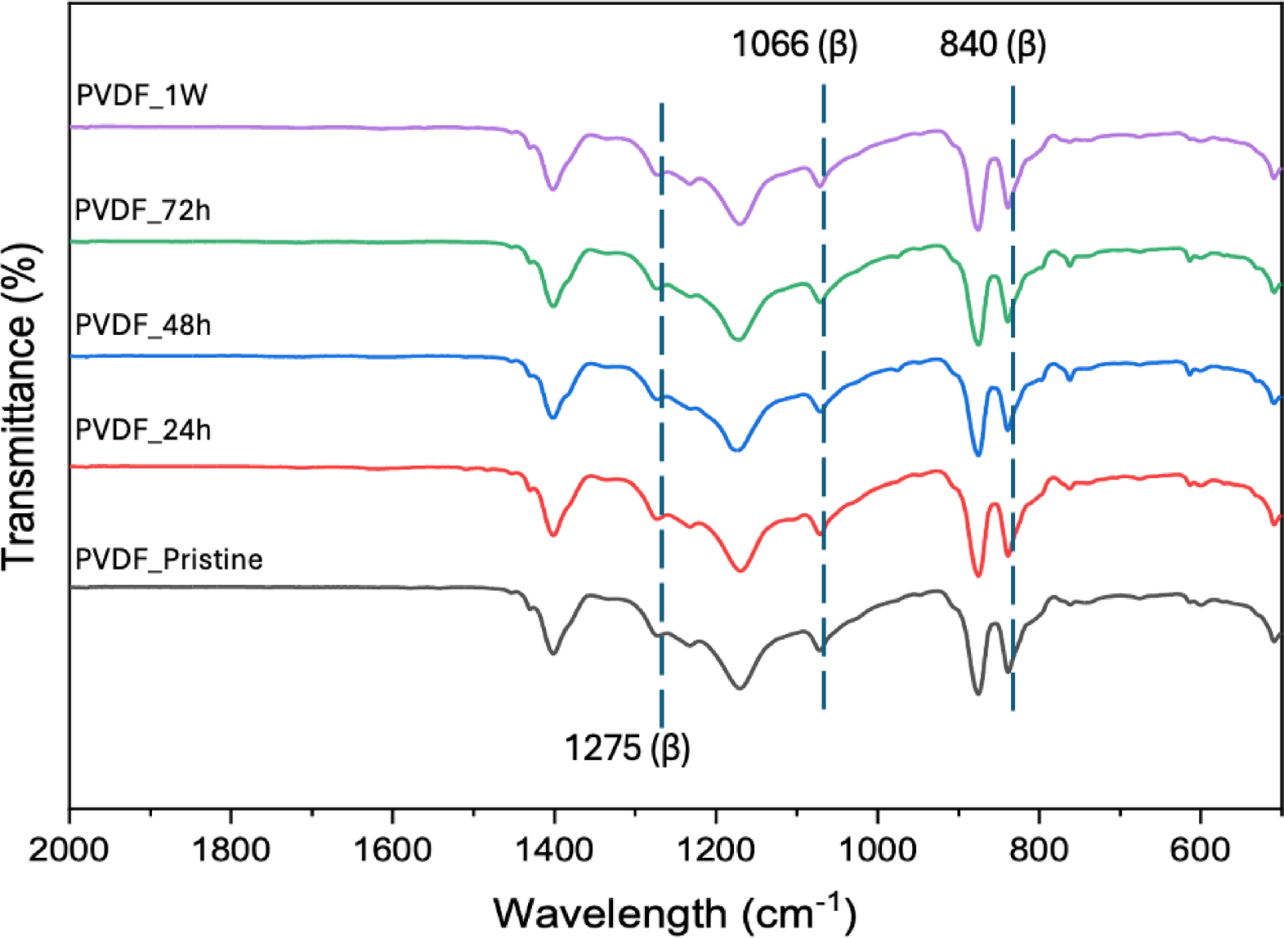
FTIR spectrum of PVDF membrane at different DES exposure time.

**Fig. 6:**
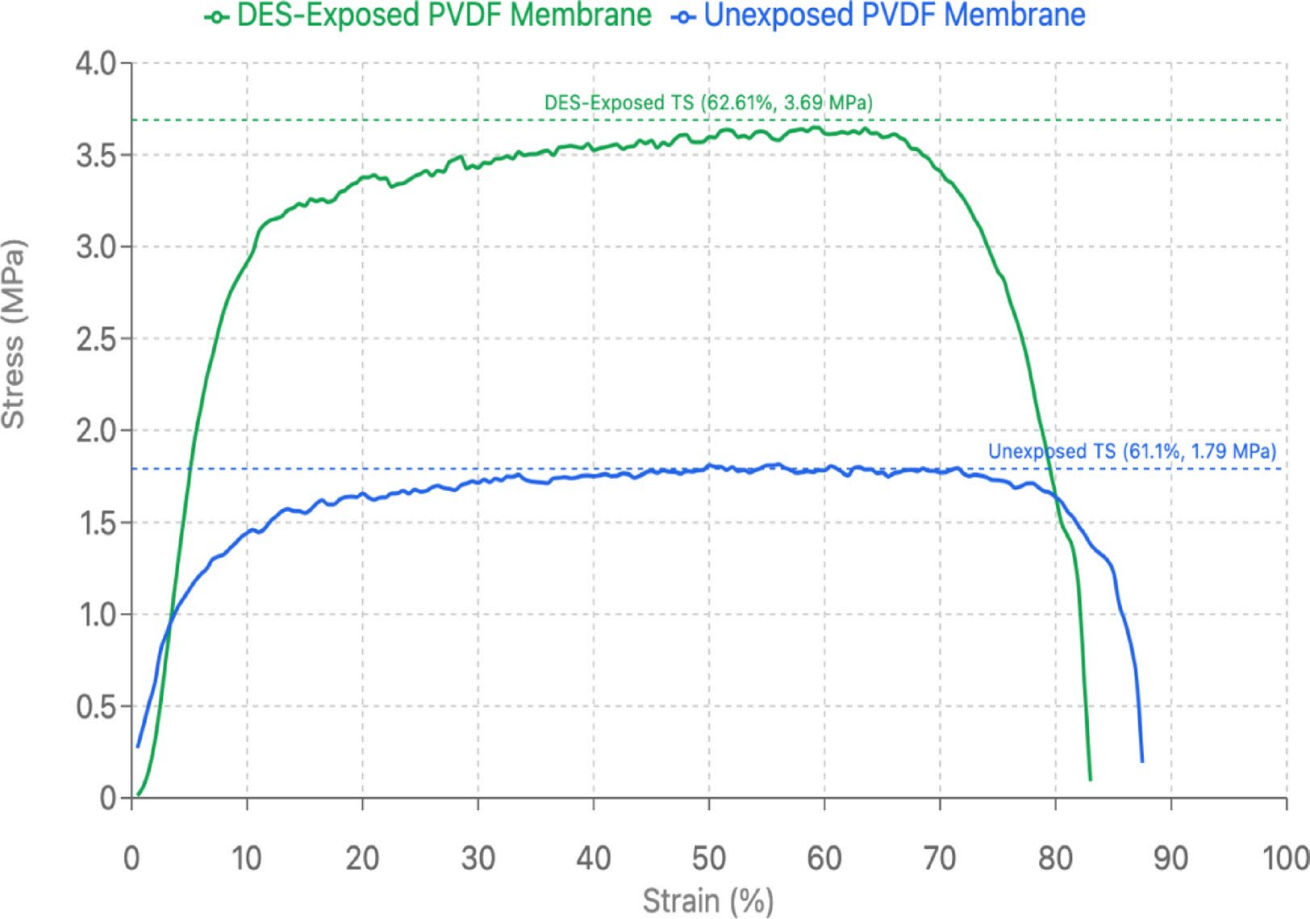
Stress-strain analysis of PVDF membrane before and after DES exposure demonstrating mechanical property changes.

Comprehensive characterization was conducted to evaluate the PVDF membrane-DES interactions and structural modifications following exposure. FTIR analysis was performed on the membrane after a duration of exposure, as shown in Fig. 5. FTIR analysis revealed characteristic peaks at 840, 1066, and 1275 cm⁻¹, confirming the presence of pure β phase PVDF with no corresponding α phase peaks detected in the membrane samples^60^. The retention of these characteristic β phase peaks after DES exposure indicates that the crystalline structure of PVDF remained unaltered, demonstrating the membrane’s chemical stability^28^. This is critical because phase transitions in PVDF can significantly affect membrane performance, and the maintained β phase structure ensures consistent filtration properties^61^. The absence of new peaks or peak shifts confirms that no chemical reactions occurred between the DES components and the PVDF backbone, validating the membrane’s chemical inertness under these conditions^18^. This structural integrity was further validated through tensile strength testing as shown in Fig. 6, where the stress-strain analysis showed that the exposed membrane exhibited enhanced tensile strength of 3.69 MPa with 62.61% strain capability, compared to the unexposed membrane’s 1.79 MPa and 61.11% strain. This increased tensile strength value, suggests that DES components act as plasticizers without compromising the structural integrity of the membrane^62^. The enhanced mechanical properties are crucial for industrial applications where membranes must withstand operational pressures and mechanical stress^27^. This increased tensile strength indicates the presence of DES on the membrane post-exposure while maintaining structural integrity with sustained strain values, which is advantageous for long-term membrane performance^63^. The mechanical enhancement correlates with the weight gain results in Fig. 3a, suggesting that residual DES components contribute positively to the membrane’s physical properties while preserving mechanical stability through maintained elongation characteristics. These FTIR and tensile results confirm that the increased mass results from physical phenomena rather than chemical modification of the polymer matrix, establishing a foundation for understanding the nature of membrane-DES interactions.

**Fig. 7:**
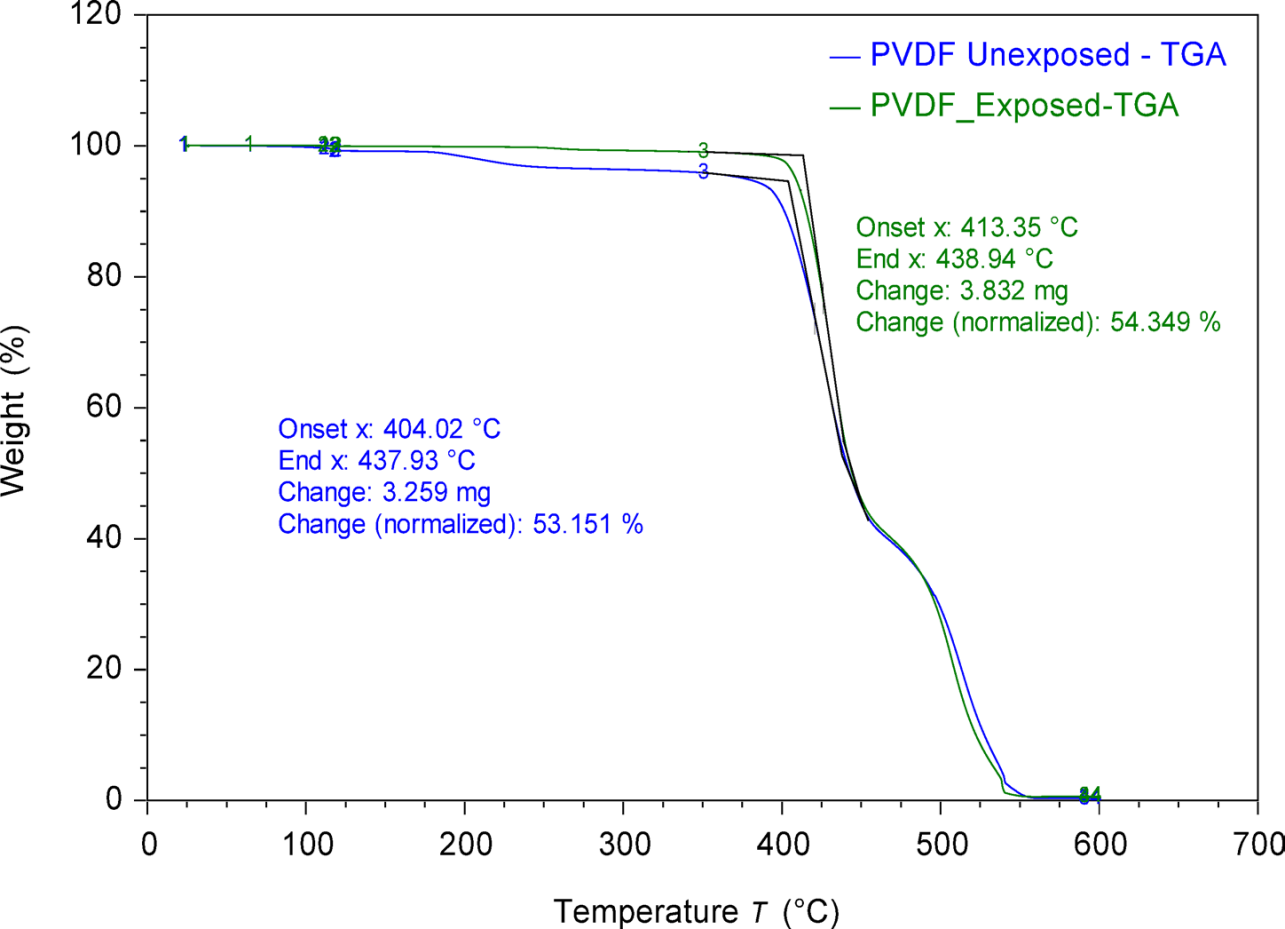
TGA thermograms of unexposed and exposed PVDF membrane to DES showing thermal degradation behavior and onset temperature profiles.

**Fig. 8:**
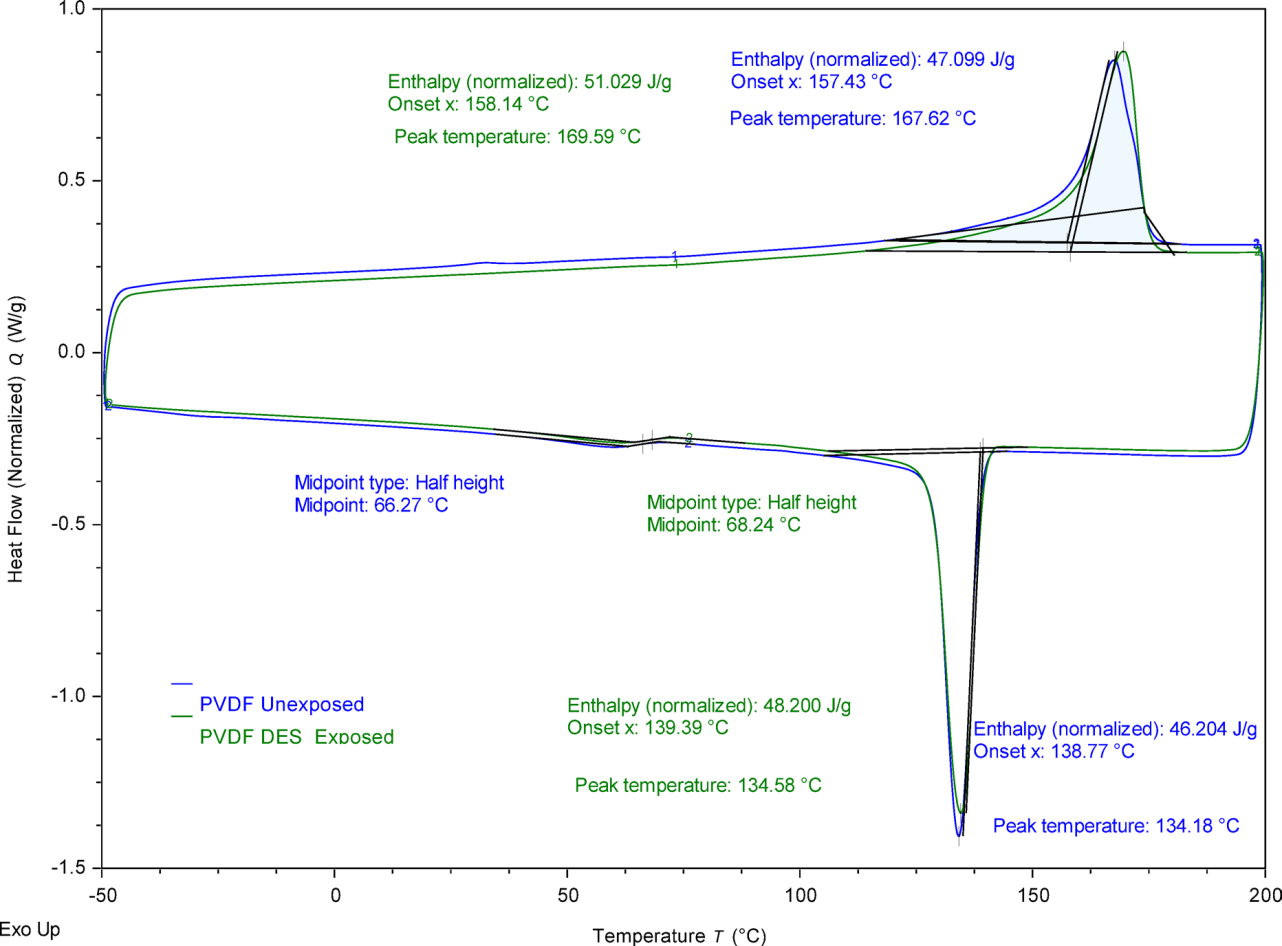
DSC thermograms of PVDF membrane pre- and post-exposure to DES showing crystallization and melting transition temperatures.

Thermal analyses were performed as shown in Figs. 7 and 8 representing the TGA and DSC analysis, respectively. TGA analysis (Fig. 7) demonstrated that the onset degradation temperature increased from 404.02 °C, which is relatively established for PVDF^64^, for unexposed membrane to 413.35 °C for the exposed membrane, while maintaining comparable normalized weight changes of 53.15% and 54.34%, respectively. This 9.33 °C increase is attributed to the presence of DES components within the membrane matrix, while confirming that the PVDF membrane maintains its thermal stability due to its inherent strong C-F bonds and semi-crystalline structure^65^. DSC characterization (Fig. 8) supported these findings by revealing minimal changes in the crystallization behavior of PVDF following DES exposure. The melting peak temperatures remained comparable at 169.59 °C for exposed versus 167.62 °C for unexposed membranes, with crystallization peak temperatures of 134.58 °C and 134.18 °C, respectively^66^. The similarity in enthalpy values and transition temperatures indicates that DES exposure does not significantly alter the crystalline structure or thermal phase transitions of PVDF^67^. These thermal properties confirm that the presence of DES components does not compromise the fundamental polymer structure. Both TGA and DSC analyses confirmed the thermal stability of PVDF following DES exposure, reinforcing the structural integrity observations from previous characterizations.

**Fig. 9:**
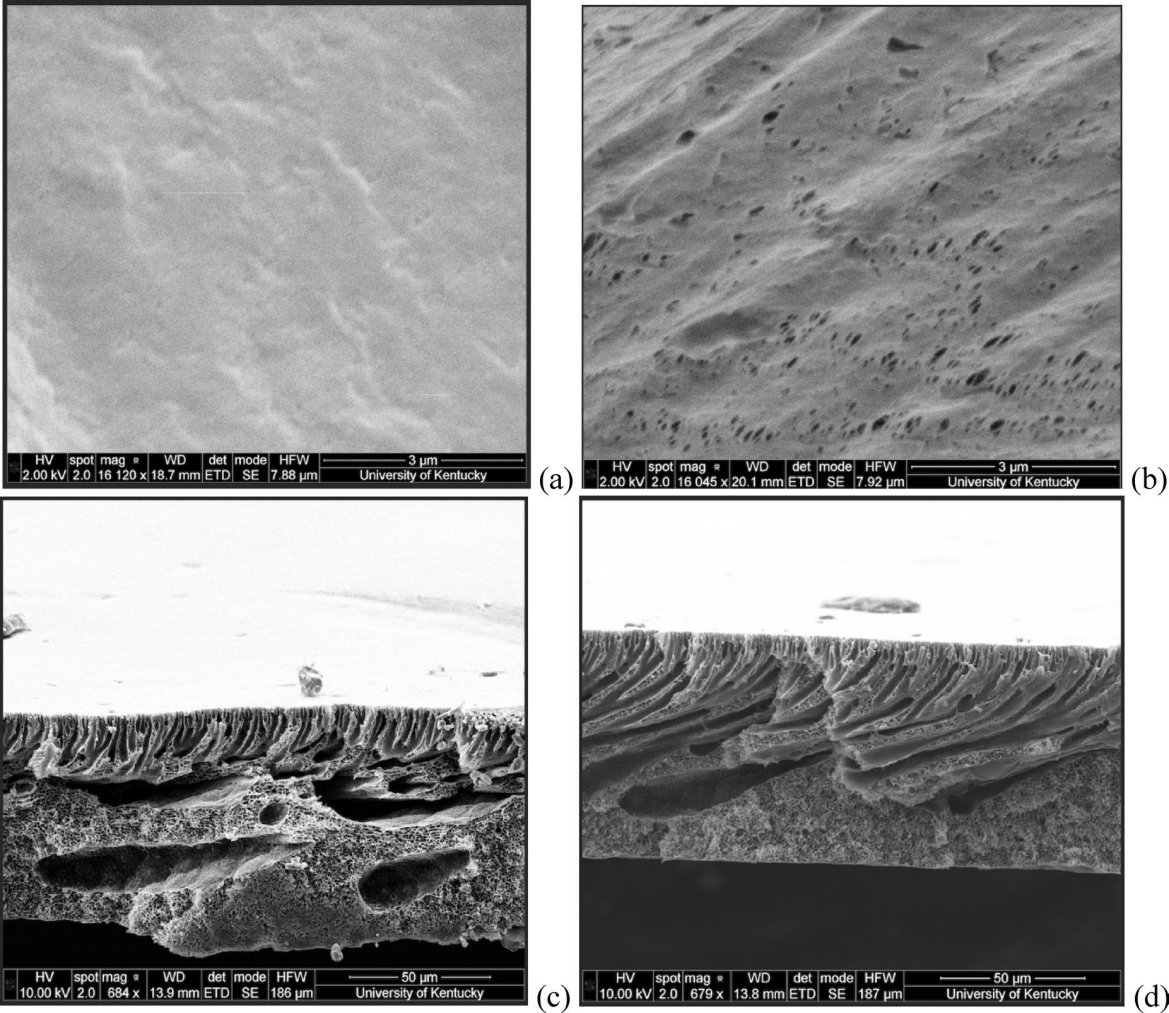
SEM images of PVDF membrane: (**a** and **c**) unexposed surface and cross-section respectively; (**b** and **d**) DES-exposed surface and cross-section respectively.

SEM analysis provided direct morphological evidence to the PVDF-DES interaction as seen in Figs. 9a–d. Surface SEM images, Figs. 9a and b, revealed evidence of DES penetration into the membrane structure, corroborating the observed weight increase as well as the wettability results from the contact angle, due to the coherent hydrophobic nature of the DES and PVDF^68^. However, cross-sectional views (Figs. 9c and d) demonstrated consistent asymmetric structure with finger-like, and sponge-like regions as seen by Nursiah et al. (2023)^69^ confirms the PVDF membrane’s resistance to degradation by the DES. The surface penetration visible in SEM are important because they indicate localized DES interaction without bulk structural compromise. This surface-selective interaction is advantageous as it allows for DES uptake without affecting the membrane’s core filtration structure.

**Fig. 10:**
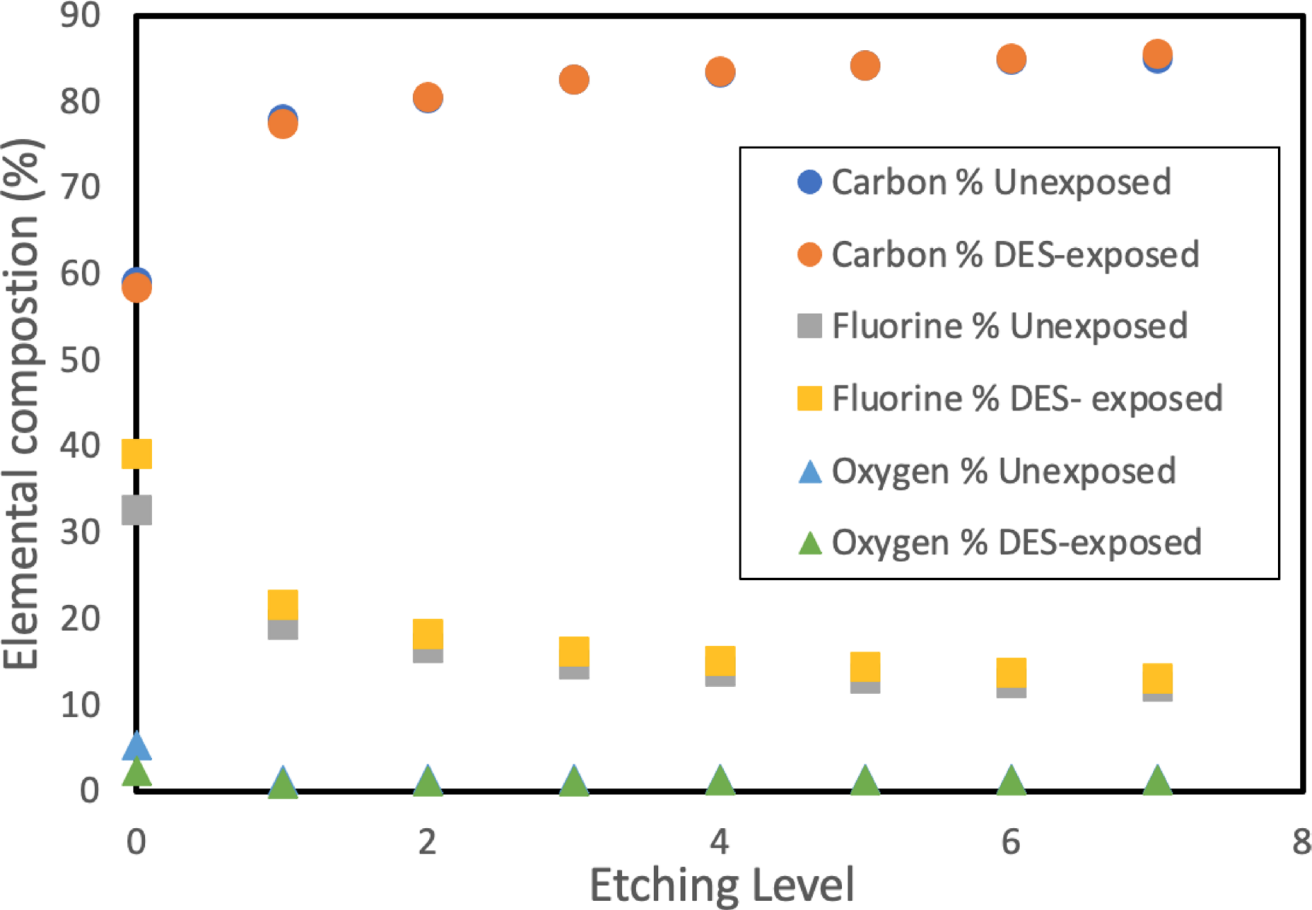
XPS depth profile analysis of unexposed and DES-exposed PVDF membrane showing elemental composition distribution across different etching levels.

**Fig. 11:**
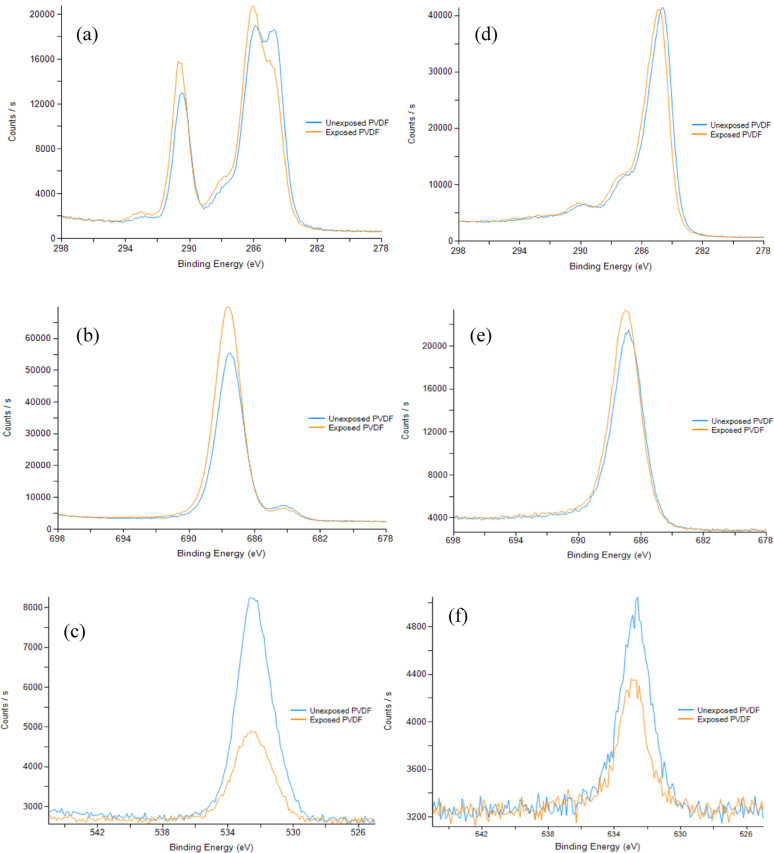
XPS analysis for elements spectra for unexposed and exposed PVDF: (**a** and** d**) are C1s; (**b** and** e**) are F1s; (**c** and** f**) are O1s at etch level 0 and 7, respectively.

XPS characterization with depth profile analysis was performed as shown in Fig. 10 highlighting the elemental percentage composition across various depths, and Figs. 11a–f elemental spectra of depth level 0 and 7. PVDF has a theoretical C/F ratio of 1:1 due to its polymer structure (–CH₂–CF₂–)n^18,28^. However, since PEG was used as a pore former as validated in the FTIR analysis showing the characteristic C–O–C asymmetric stretching vibration at 1240–1250 cm⁻¹ and C-H rocking vibrations at 840–960 cm⁻¹ associated with PEG, as shown in Figure S2 of the supplementary section, the C/F ratio deviated from unity. The FTIR peaks observed in the PVDF membrane with PEG pore former confirm the presence of residual PEG in the membrane matrix, as complete removal of PEG during the phase inversion process is usually unattainable, and some residues remain trapped in the membrane matrix^70^. The XPS depth profiling results revealed significant compositional changes between unexposed and DES-exposed membranes. As shown in Fig. 10, fluorine content increased from 32.61 to 39.14% at etch level 0, while oxygen content decreased dramatically from 5.36 to 2.43% at the surface. Notably, carbon content remained relatively unchanged (59.06% vs. 58.43%), indicating that the DES is selectively extracting oxygen-rich PEG from the surface while simultaneously adsorbing carbon-rich aromatic compounds (Thymol and 2,6-dimethoxyphenol) to the membrane surface. While PBI membranes showed weight loss indicating membrane leaching, PVDF membranes demonstrated this different behavior, where the residual PEG (pore-former) was selectively extracted from the membrane surface while DES components were adsorbed. The unequal C/F ratio persisted throughout the bulk material (etch levels 0–7), confirming that PEG extraction occurred primarily at the surface with diminishing effects at greater depths. This simultaneous extraction and adsorption mechanism explains the net weight gain observed in Fig. 3a.

The elemental spectra analysis in Figs. 11 provides further evidence for this mechanism. The C1s spectra (Fig. 11a and d) show distinct binding energy peaks at approximately 285 eV (C–C/C–H bonds), 286.5 eV (C–O bonds characteristic of PEG), and 291 eV (CF₂ bonds from PVDF)^71,72^. At etch level 0, the exposed sample exhibited reduced intensity in the C–O peak region compared to the unexposed sample, confirming PEG extraction, while showing increased C–C/C–H peak intensity, indicating DES component adsorption. The fluorine spectra (Fig. 11b and e) demonstrate the characteristic F1s peak at approximately 688 eV^73^, with higher intensity in the exposed sample at the surface. The oxygen spectra (Fig. 11c and f) at 532 eV^74^ clearly show the dramatic reduction in oxygen content after DES exposure, particularly at the surface level.

These findings demonstrate that DES components primarily interact at the membrane surface through adsorption mechanisms rather than bulk penetration. The surface-dominated mechanism necessitates detailed adsorption kinetic and isotherm studies to quantify and predict membrane-DES interactions for process optimization.

### Adsorption mechanisms

The adsorption behavior of deep eutectic solvents (DES) by PVDF membranes was investigated under various experimental conditions as outlined in Table 1. To accurately measure concentration differences after exposure duration, the DES was dissolved in n-hexane as a carrier solvent. The choice of n-hexane as a solvent carrier was critical due to solubility of the DES in it^9,33^. Analysis of the experimental data revealed that adsorption mechanisms were only observed at 15 °C with 80v/v% DES concentration, this selective adsorption behavior was attributed to favored n-hexane evaporation or no significant adsorption experienced in other experimental conditions as shown in Figures S3-S5 of the supplementary section. The presence and behavior of n-hexane under varying temperature and concentration conditions significantly influenced the adsorption dynamics due to its low evaporation temperature and volatility characteristics^75,76^, making it competiting with the DES for adsorption sites. Therefore, the 15 °C and 80 v/v% condition was selected for detailed kinetics investigation, as it provided the most suitable experimental conditions for studying DES-membrane interactions without interference from n-hexane.

### Kinetics model

**Fig. 12:**
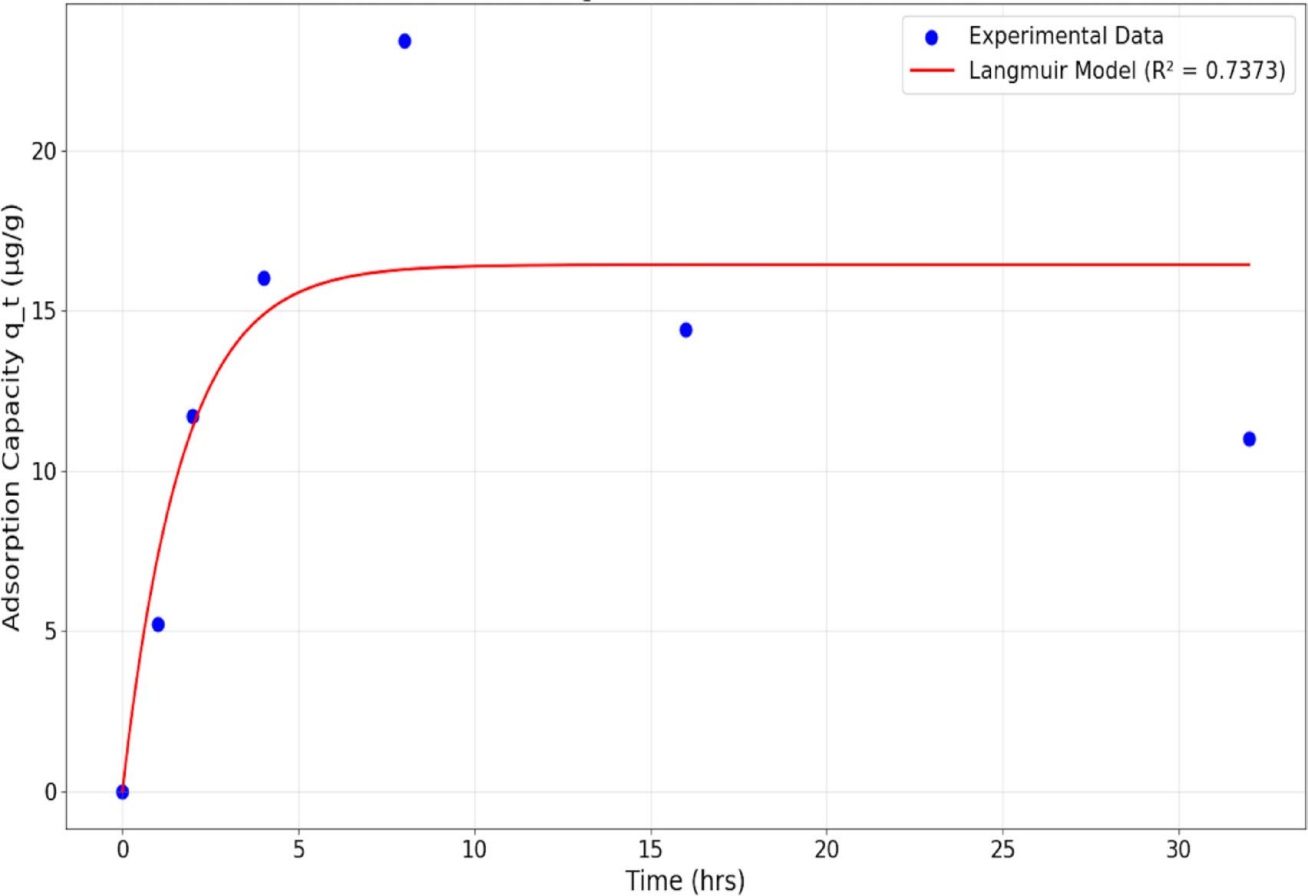
Langmuir adsorption kinetic model fitting to experimental data showing theoretical curve correlation with observed DES adsorption behavior on PVDF membrane.

The observed adsorption mechanism exhibited an initial adsorption phase followed by desorption, characteristic of complex adsorption-desorption equilibrium systems^35^. To understand the underlying kinetics, the experimental data was first modeled using the Langmuir adsorption kinetics framework, as presented in Fig. 12. The Langmuir kinetics approach provides insight into the fundamental rate processes governing adsorption and desorption at the membrane-DES interface^36,70^. The Langmuir kinetic model can be expressed as a hybrid equation that incorporates both first order and second-order components^35,36^. The relative dominance of these components depends on the relationship between the equilibrium adsorption capacity (q_e_) and the ratio of rate constants (k₁/k₂)^77^. When q_e_ ≪ k₁/k₂, the hybrid equation simplifies to the pseudo-first order (PFO) model, signifying that PFO kinetics best explains the entire system behavior. Conversely, when q_e_ approaches or exceeds k₁/k₂, the system tends toward pseudo-second order (PSO) behavior^78–80^. From the Langmuir kinetics analysis, the following parameters were determined: R² = 0.7373, k₁ = 0.5888 h⁻¹, k₂ = 0.000010 (µg/g) ⁻¹ hr⁻¹, q_e_ = 16.4363 µg/g, and k₁/k₂ = 58,879.26 µg/g. The critical comparison reveals that q_c_ (16.4363 µg/g) ≪ k₁/k₂ (58,879.26 µg/g), indicating that the model effectively reduces to pseudo-first-order kinetics. This result demonstrates that PFO dominates the kinetics in this system, suggesting that the adsorption rate is primarily controlled by the concentration gradient and the availability of adsorption sites^81,82^.

**Fig. 13:**
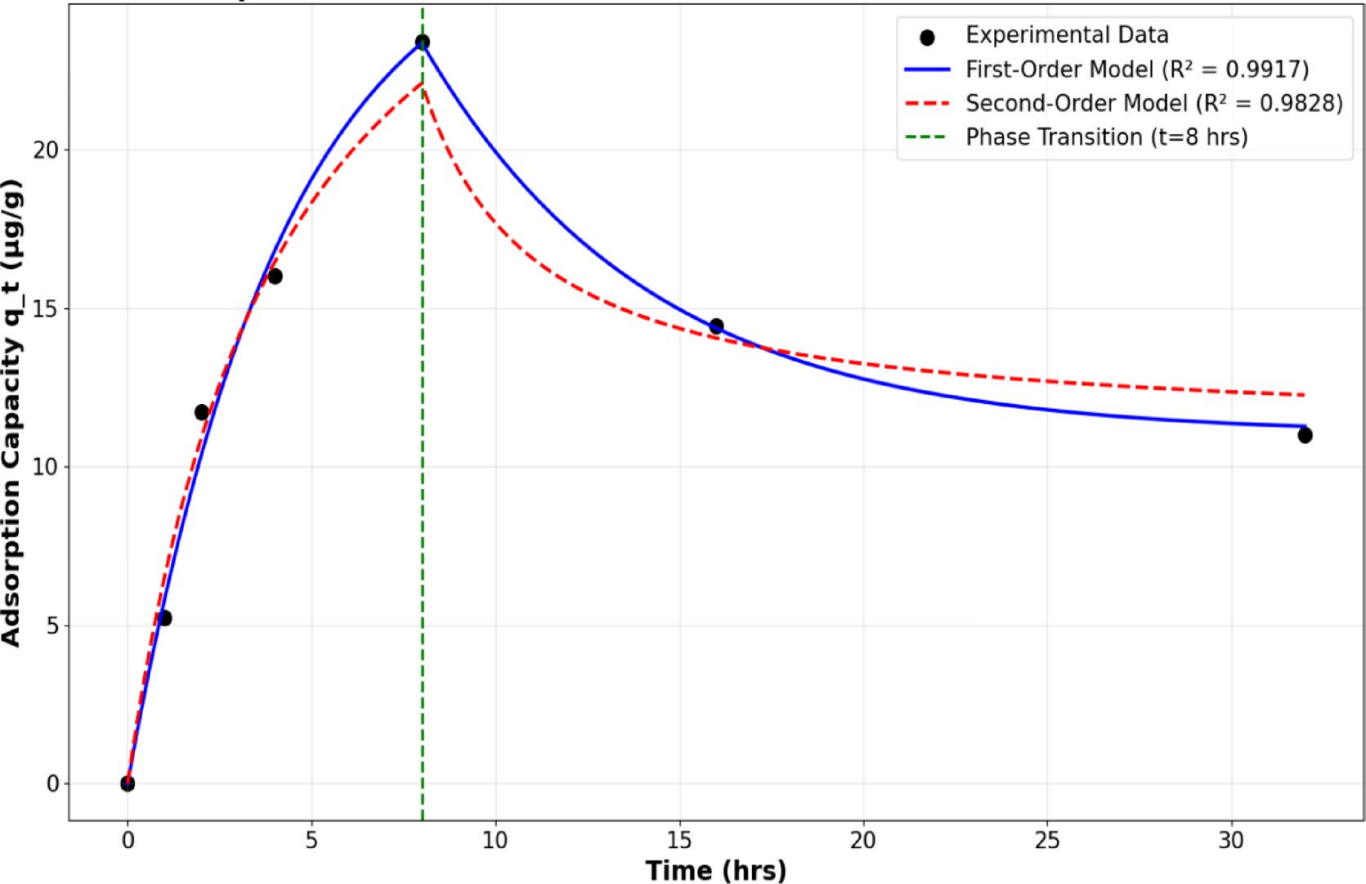
Reversible pseudo first-order and second-order kinetic model fitting to experimental data showing comparative analysis of adsorption mechanisms.

To validate this conclusion and compare kinetic models, the experimental data was fitted to both reversible first order and second-order models, as illustrated in Fig. 13. The reversible first-order model showed superior correlation with the experimental data, exhibiting a higher R² = 0.9917 value compared to the reversible second-order model (R² = 0.9828). Complete kinetic model parameters are provided in Supplementary Table S1. This confirms that the DES adsorption onto PVDF membranes follows predominantly first-order kinetics, where the rate-limiting step involves the initial interaction between DES molecules and membrane surface sites^83,84^.

The reversible nature of the adsorption process, evidenced by the initial adsorption followed by desorption, suggests that the DES-membrane interactions are primarily physical rather than chemical in nature. This behavior is consistent with the temporary nature of physical adsorption^85^ and indicates that the membrane surface does not permanently bind DES molecules, allowing for potential membrane regeneration.

### Isotherm model

To further characterize the adsorption behavior and determine the equilibrium relationships between DES concentration and adsorption capacity, various isotherm models were applied to the experimental data. The classical Langmuir, Freundlich, and Temkin isotherm models were evaluated to identify the best-fitting model for the DES-PVDF membrane system as shown in supplemental section Figures S6, S7 and S8 respectively. The isotherm parameters and correlation coefficients for each model are presented in Table 4. The Langmuir isotherm model, which assumes monolayer adsorption on homogeneous surfaces with finite adsorption capacity, showed limited applicability to this system with R² = 0.018. The negative values for both the Langmuir constant (K_d_ = − 160995 L/mg) and maximum adsorption capacity (q_m_ = − 5000 mg/g) indicate that the fundamental assumptions of the Langmuir model are not met for this adsorption system^86,87^. In contrast, the Freundlich isotherm model demonstrated excellent correlation with the experimental data, achieving R² = 0.9983. The Freundlich parameters (K = 4.5563 L/mg and *n* = 1.0082) indicate favorable adsorption characteristics, with the n value very close to unity suggesting near-linear adsorption behavior over the concentration range studied. The high correlation coefficient confirms that the DES adsorption follows multilayer adsorption on heterogeneous surfaces, which is consistent with the complex surface morphology of PVDF membranes^88^. Lastly, the Temkin isotherm model also showed excellent agreement with the experimental data, yielding R² = 0.9987, which represents the highest correlation among all tested models. The Temkin parameters (A = 5.6086 and B = 0.043) suggest that the adsorption process involves significant adsorbent-adsorbate interactions and that the heat of adsorption decreases linearly with surface coverage^46,82,89^. The superior fit of the Temkin model indicates that the adsorption is characterized by uniform distribution of binding energies and considers the effects of indirect adsorbate-adsorbate interactions^90,91^.

**Table 4:** Fitting experimental data to isotherm models parameters.

Isotherm Model	*R*-squared value	Parameters	
		K_d_ (L/mg)	q_m_
Langmuir	0.018	− 160,995	− 5000
Freundlich	0.9983	*K (L/mg)*	*n*
		4.5563	1.0082
Temkin	0.9987	*A*	*B*
		5.6086	0.043

Based on the correlation coefficients, the Temkin isotherm model provides the best description of the DES adsorption equilibrium, followed closely by the Freundlich model. Both models significantly outperform the Langmuir model, indicating that the adsorption process involves heterogeneous surface interactions rather than simple monolayer coverage. The excellent fit of the Temkin model suggests that the adsorption energy distribution and interaction effects play crucial roles in determining the equilibrium adsorption capacity^92–94^.

### Membrane performance analysis: permeability and rejection

**Fig. 14:**
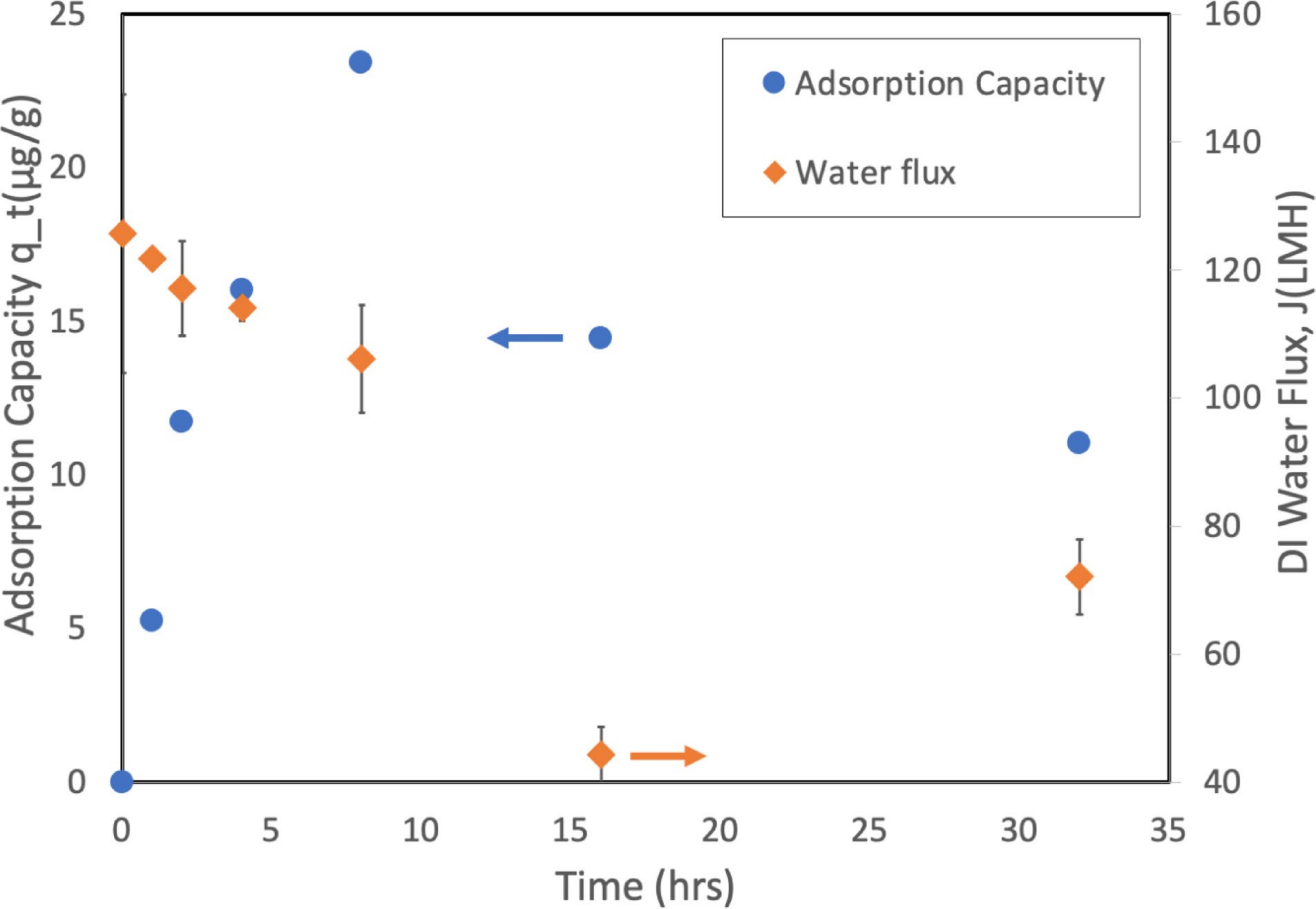
Relationship between membrane adsorption capacity of DES and pure water flux during membrane adsorption experiments.

The relationship between membrane adsorption capacity of DES and pure water flux performance is presented in Fig. 14, which demonstrates how both parameters changed throughout the adsorption experiment. This inverse relationship between adsorption capacity and flux confirms that membrane adsorption behavior as similarly reported by Zhang et al.^46^, providing further evidence supporting the adsorption mechanisms identified in “Adsorption mechanisms” section. The flux partially recovering to about 72 LMH at 32 h, indicating the adsorption-desorption nature of DES-membrane interactions.

**Fig. 15:**
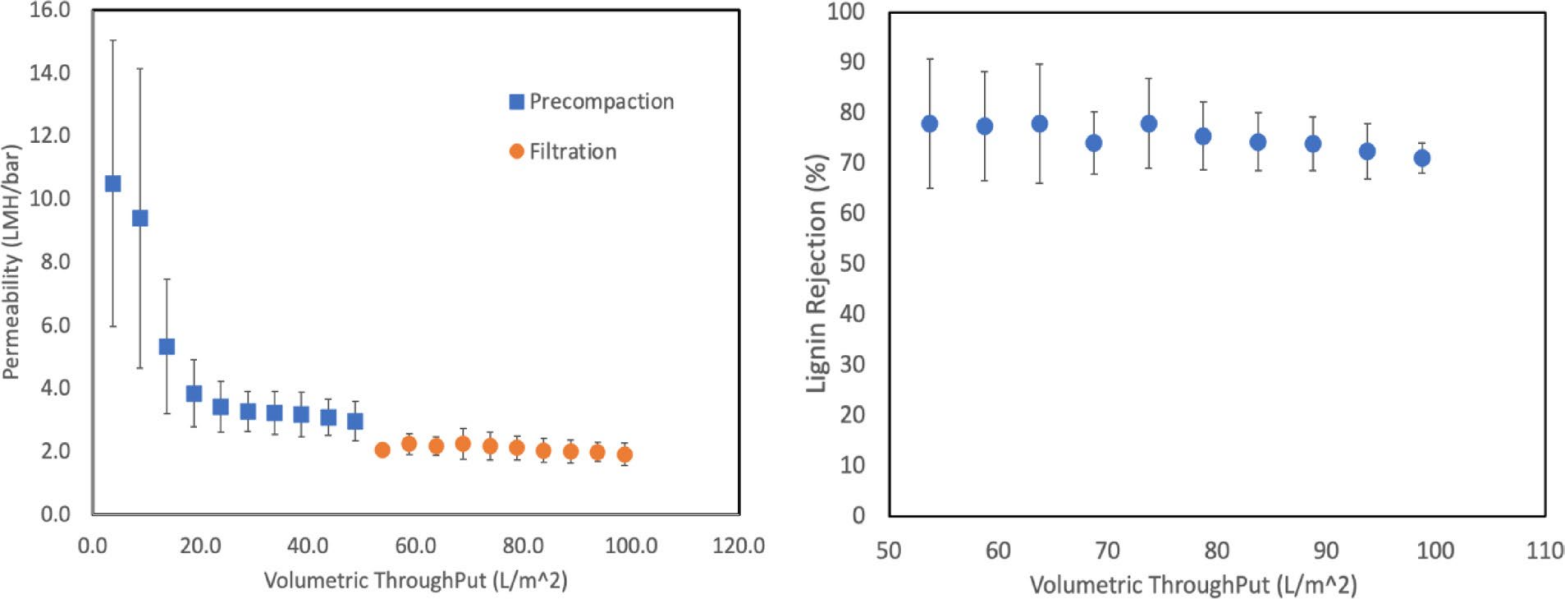
Membrane performance evaluation: (**a**) DES permeability during pre-compaction and filtration phases and (**b**) lignin rejection efficiency.

Membrane performance was investigated through the DES permeability and Lignin filtration phases are illustrated in Figs. 15a and b, showing the volumetric throughput versus permeability relationship. During the pre-compaction phase, initial permeability values ranged from 10.5 to 9.5 LMH/bar with considerable variability, reflecting the membrane conditioning process and establishment of stable operating conditions^95–97^. During the filtration phase, permeability values further decreased and stabilized around 2.0 LMH/bar, demonstrating the impact of DES interaction on membrane transport characteristics and confirming the fouling of the membrane due to lignin rejection^98–100^.

Lignin rejection performance as a function of volumetric throughput is presented in Fig. 15b, demonstrating the membrane’s separation efficiency throughout the filtration process. Initial lignin rejection values were approximately 77%, with rejection efficiency remaining relatively stable throughout the experiment, consistently ranging between 72% and 77%. The slight decline in rejection efficiency from 77 to 72% over the course of filtration can be attributed to membrane fouling by the lignin^33,101,102^, however, the overall stability demonstrates that the PVDF membrane maintains its structural integrity and separation functionality under the tested conditions. The consistent rejection values indicate that the primary separation mechanism remains size exclusion, with the membrane exhibiting suitable characteristics for DES-lignin separation applications^96,103–105^.

The comparison of membrane performance for DES applications demonstrates the variability in material selection and processing conditions across different studies. While direct comparisons are challenging due to varying experimental conditions, DES compositions, and membrane fabrication materials, this benchmarking provides valuable context for this study as shown in Table 5.

**Table 5:** Performance comparison of PVDF membranes with other membranes for DES recovery from lignin.

Membrane material	Membrane type	DES name	DES permeability (LMH/bar)	Lignin rejection (%)	References
Polyvinylidene fluoride	Hydrophobic	Thy: Dmp	2.0 ± 0.34	75.2 ± 7.69	This study
Polyethersulfone (commercial)	Hydrophobic	ChCl: Lactic acid	1.5–2.5	85	^101^
Cellulose (commercial)	Hydrophilic	ChCl: Lactic acid	1.15	70	^106^
Functionalized polyimide	Hydrophobic	ChCl: Lactic acid	2.36–8.28	44-66.4	^104^

The benchmarking analysis reveals that PVDF membranes demonstrate competitive performance characteristics within the range of existing membrane technologies for DES-lignin separation applications. The permeability values obtained (2.0 ± 0.34 LMH/bar) fall within the intermediate range compared to other materials, while achieving lignin rejection performance (75.2 ± 7.69%) that is superior to some technologies and comparable to others as seen in Table 5. Notably, this study represents one of the few investigations utilizing hydrophobic DES systems, as most reported studies focus on hydrophilic ChCl-based DES formulations. The hydrophobic nature of both the Thy: Dmp DES and PVDF membrane combination offers distinct advantages for water-immiscible processing applications, while the systematic HSP-based material selection approach provides a rational framework for predicting membrane-solvent compatibility in emerging DES systems.

## Conclusion

This study established a systematic methodology for membrane material selection in hydrophobic deep eutectic solvent applications using the Hansen Solubility Parameter approach with inverted criteria. PVDF membranes demonstrated compatibility with lignin-derived hydrophobic DES through maintained chemical, thermal and mechanical stability following DES exposure validated by the various characterization techniques. The membrane-DES interaction mechanism was identified as surface-selective physical adsorption following pseudo-first-order kinetics with reversible characteristics, as confirmed by XPS depth profiling showing simultaneous PEG extraction and DES component adsorption at the membrane surface. The Temkin isotherm model best described the equilibrium behavior (R² = 0.9987), indicating heterogeneous surface interactions. PVDF membranes-maintained separation functionality with stable lignin rejection of 77 − 72% and demonstrated flux recovery capabilities, confirming the reversible nature of membrane-solvent interactions. Future studies should include extended exposure testing over 3–6-month periods, evaluation of membrane regeneration strategies, and assessment of cumulative fouling effects during repeated filtration cycles. These investigations are essential for establishing the practical viability of PVDF membranes in industrial DES processing applications and would provide comprehensive data on membrane durability under continuous operational conditions. This work provides a framework for rational membrane selection in emerging solvent systems, contributing to the body of knowledge of sustainable DES-based biomass separation technologies and applications.

## Supplementary Information

Below is the link to the electronic supplementary material.

Supplementary Material 1.

Supplementary Material 2.

## Data Availability

All datasets generated and/or analysed during the current study are available in a LabArchives repository: https://drive.google.com/drive/folders/1ijCDJuQP7PfhotsTT6CbNcB5oTOIIhIk? usp=sharing.
