# Supplementary material for: Adsorptive behavior of poly (vinylidene fluoride) membranes for the recovery of lignin-derived hydrophobic deep eutectic solvents
Source: Sci Rep. 2025 Sep 1;15:32051. doi: 10.1038/s41598-025-18164-x (PMC12402438; doi:10.1038/s41598-025-18164-x)
Supplement: Supplementary file 1 — Supplementary Material 1. [file 41598_2025_18164_MOESM1_ESM.docx]

**﻿*Supplementary Information***

Adsorptive Behavior of Poly (vinylidene fluoride) Membranes for the Recovery of Lignin-derived Hydrophobic Deep Eutectic Solvents

Odianosen I. Ewah^1^, ﻿Yuxuan Zhang^2^, Jian Shi^2^, & Isabel C. Escobar^*^

^1.^ ﻿Department of Chemical and Materials Engineering, University of Kentucky, Lexington, KY 40506, USA; [Odianosen.Ewah@uky.edu](mailto:Odianosen.Ewah@uky.edu)

^2.^ ﻿Biosystems and Agricultural Engineering, 128 C.E. Barnhart Building, University of Kentucky, Lexington, KY, 40506, USA; asm.sayem@uky.edu; [yx.zhang@uky.edu](mailto:yx.zhang@uky.edu); j.shi@uky.edu

* ﻿ ﻿Correspondence: [isabel.escobar@uky.edu](mailto:isabel.escobar@uky.edu); Tel.: +1-859-257-7990

**List of Abbreviations**

- DES: Deep Eutectic Solvent
- HDES: Hydrophobic Deep Eutectic Solvent
- PVDF: Polyvinylidene Fluoride
- PBI: Polybenzimidazole
- PSf: Polysulfone
- HSP: Hansen Solubility Parameters
- RED: Relative Energy Difference
- NIPS: Non-solvent Induced Phase Separation
- PEG: Polyethylene Glycol
- FTIR: Fourier Transform Infrared Spectroscopy
- XPS: X-ray Photoelectron Spectroscopy
- SEM: Scanning Electron Microscopy
- TGA: Thermogravimetric Analysis
- DSC: Differential Scanning Calorimetry
- HBA: Hydrogen Bond Acceptor
- HBD: Hydrogen Bond Donor
- ChCl: Choline Chloride
- NMP: N-methyl-2-pyrrolidone
- DMF: N,N-dimethylformamide
- DMAc: N,N-dimethylacetamide
- Thy: Thymol
- Dmp: 2,6-dimethoxyphenol

SI1: MEMBRANE SOLUBILITY AND STABILITY ASSESSMENT

Figure S1 shows the visual confirmation of membrane stability following deep eutectic solvent exposure. PVDF and PBI membranes maintained their structural integrity throughout the 7-day exposure period, demonstrating no visible dissolution or degradation. This visual assessment supports the Hansen Solubility Parameter predictions where RED values greater than 1.0 indicated chemical resistance.


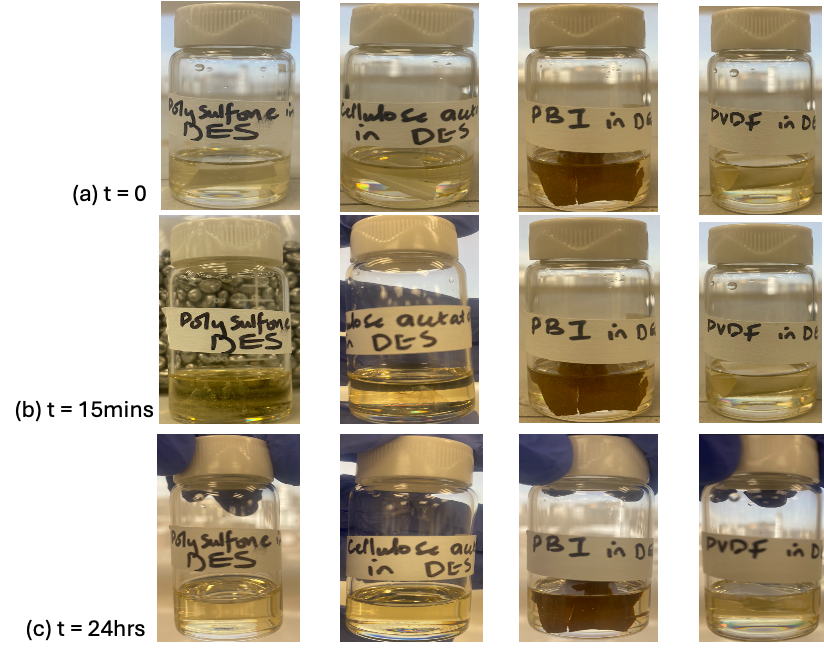


**Figure S1.** Visual assessment of the different polymeric membranes stability in DES at various time intervals: (a) initial exposure, (b) 15 minutes exposure, and (c) 24 hours exposure.

SI2: MEMBRANE CHEMICAL CHARACTERIZATION

Figure S2 presents the FTIR analysis demonstrating the presence of polyethylene glycol (PEG) pore-forming agent in the PVDF membrane structure. The spectrum shows characteristic C-O-C asymmetric stretching vibration at 1240-1250 cm⁻¹ and C-H rocking vibrations at 840-960 cm⁻¹ associated with PEG. These peaks confirm the incorporation of PEG during membrane fabrication and validate the XPS findings regarding PEG extraction during DES exposure.


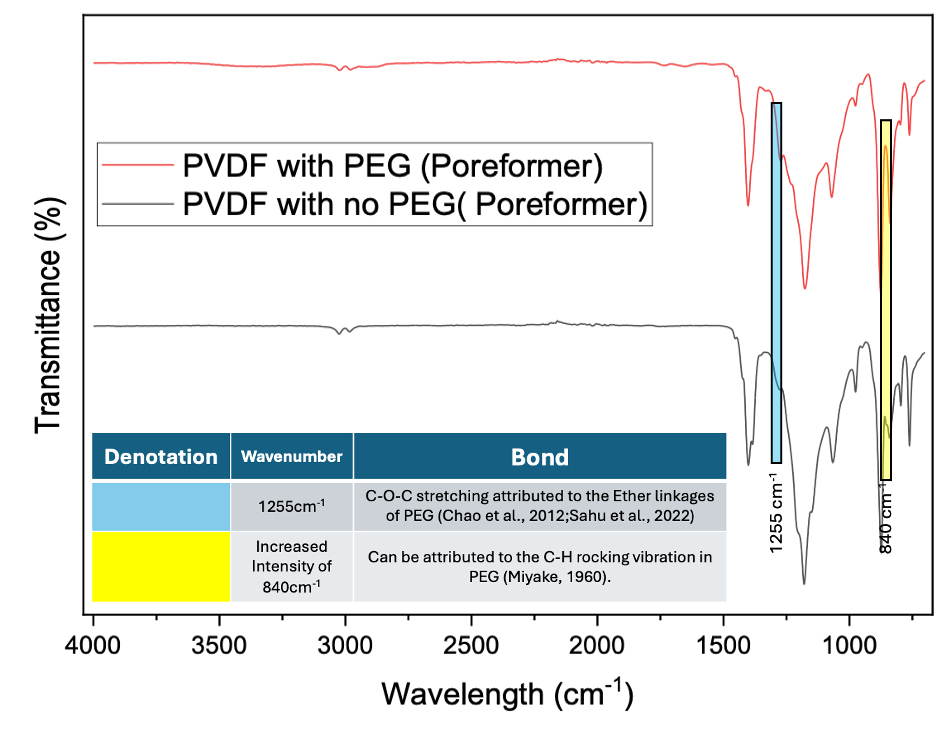


**Figure S2.** FTIR spectroscopic analysis of PVDF membrane with and without polyethylene glycol pore-forming agent showing characteristic peak differences.

SI3: DES-MEMBRANE INTERACTION UNDER VARIOUS EXPERIMENTAL CONDITIONS

Figures S3-S5 illustrate the DES concentration profiles on PVDF membranes under different experimental conditions where no significant membrane-solvent interaction was observed. These figures demonstrate the temperature and concentration dependence of the membrane-DES interaction mechanism, providing crucial insights into the selectivity of the adsorption process. The experimental conditions explored include various temperature ranges and DES concentrations to establish the adsorption window for effective membrane-solvent interactions. The absence of significant interaction under some of these specific conditions highlights the critical role of temperature and concentration in governing the membrane-DES system behavior and confirms that meaningful adsorption occurs only under very specific conditions.


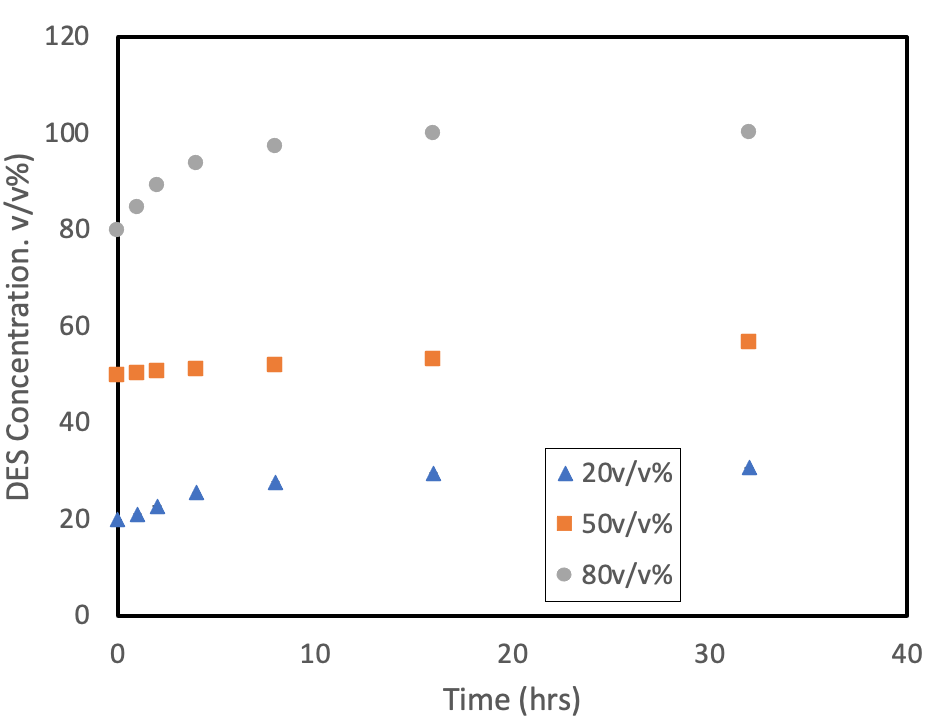


**Figure S3.** DES concentration profiles over time at 40°C showing different initial concentrations (20, 50, and 80 v/v%).


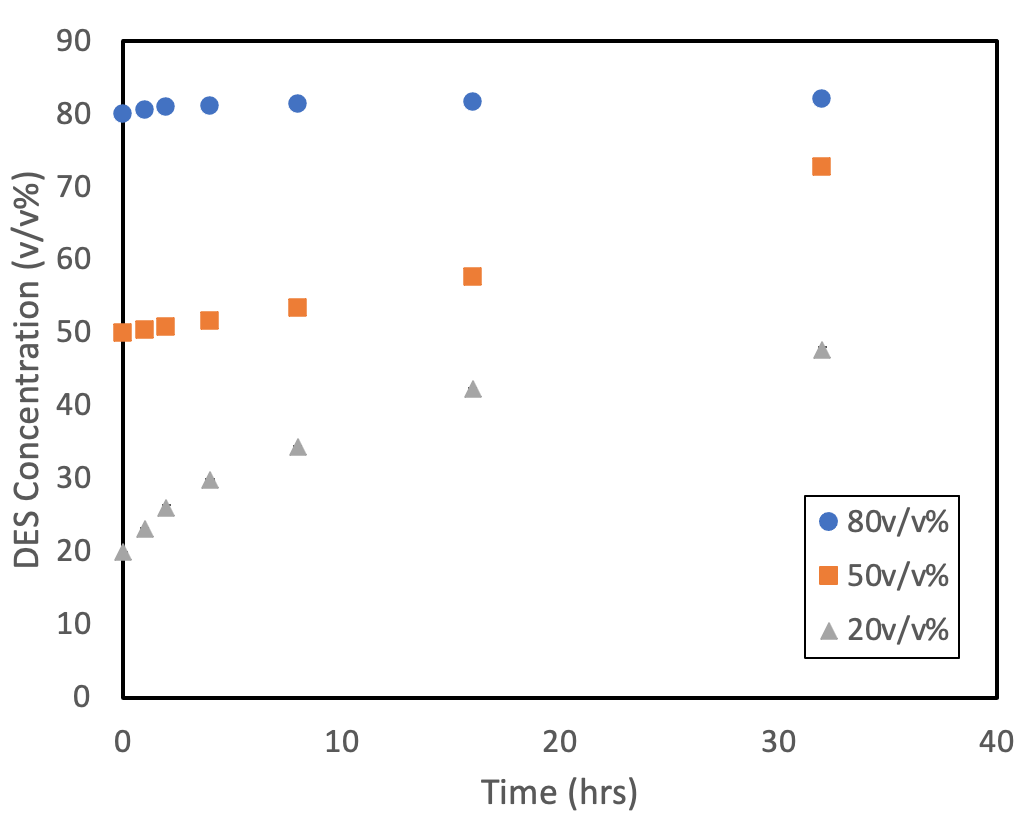


**Figure S4.** DES concentration profiles over time at room temperature (25°C) showing different initial concentrations (20, 50, and 80 v/v%).


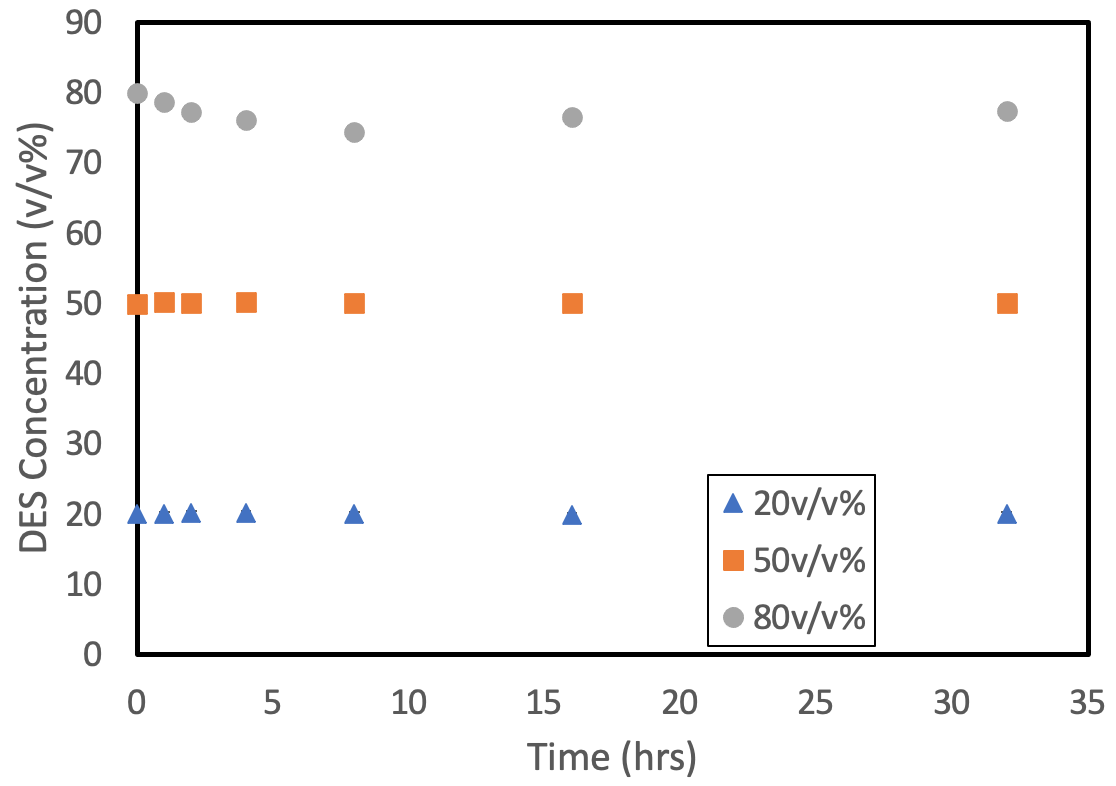

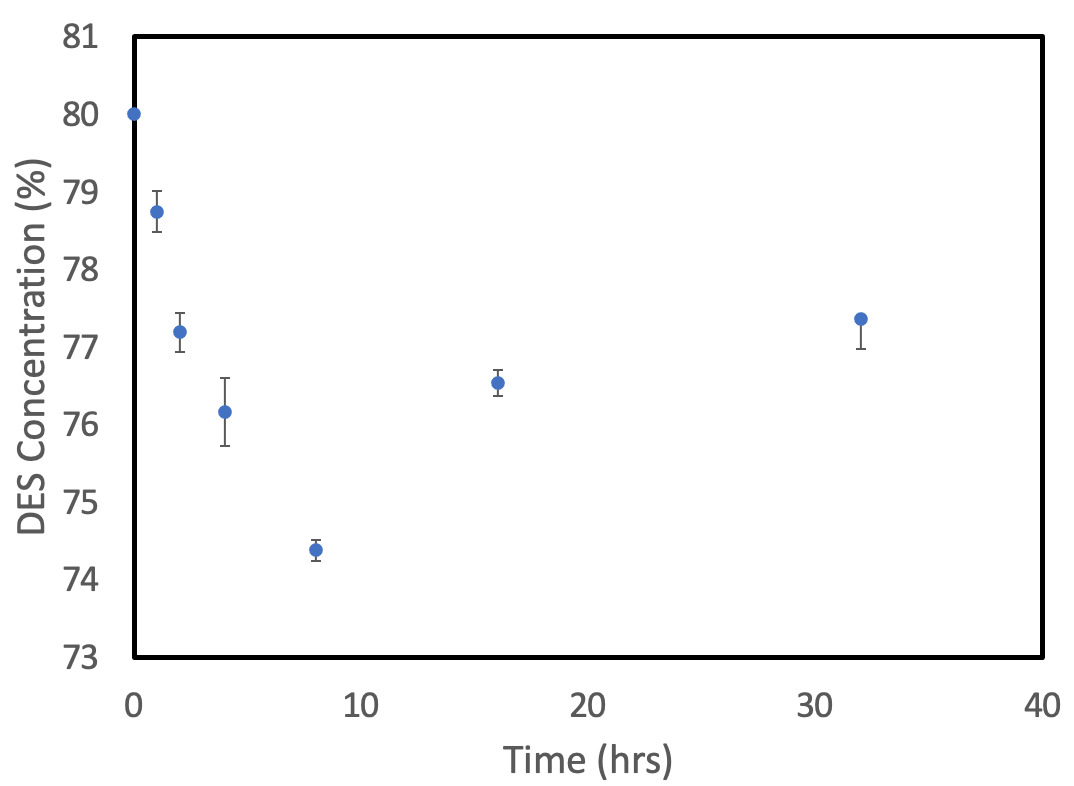


(b)

(a)

**Figure S5.** DES concentration profiles over time at 15°C showing: (a) different initial concentrations (20, 50, and 80 v/v%) and (b) adsorption behavior at 80 v/v% concentration.

The absence of significant adsorption under these conditions highlights the specificity of the interaction mechanism observed at 15°C with 80 v/v% DES concentration, demonstrating that the adsorption process is highly dependent on both temperature and concentration parameters.

SI4: KINETICS AND ISOTHERM MODEL ANALYSIS

Table S1 provides a comprehensive summary of the kinetic model parameters for DES adsorption onto PVDF membranes. The reversible pseudo-first order (PFO) model demonstrated superior correlation with experimental data compared to the reversible pseudo-second order (PSO) model. The kinetic parameters include adsorption rate constant (K₁) and desorption rate constant (K₂), which govern the forward and reverse reaction rates respectively. The table also presents the maximum adsorption capacity (Qₘ), representing the theoretical maximum amount of DES that can be adsorbed by the membrane, and the equilibrium capacity (qₘ_rev), which indicates the actual adsorption capacity achieved under reversible conditions. Both models show identical equilibrium capacities despite different kinetic behaviors, confirming the reversible nature of the DES-membrane interaction.

Table S1. Kinetic Model Parameters for DES Adsorption onto PVDF Membranes

| Kinetics Model | R-squared value | Constants | | | |
| --- | --- | --- | --- | --- | --- |
|  |  | K_1_ | K_2_ | Q_m_(µg/g) | q_m_rev_(µg/g) |
| Reversible Pseudo First Order (PFO) | 0.9917 | 0.2355hr^-1^ | 0.1632hr^-1^ | 27.57 | 11.02 |
| Reversible Pseudo Second Order (PSO) | 0.9828 | 0.0071g/µg.hr | 0.0299 g/µg.hr | 33.63 | 11.02 |

Figures S6-S8 present the isotherm model analysis for DES adsorption onto PVDF membranes, fitting of experimental data to classical adsorption models.


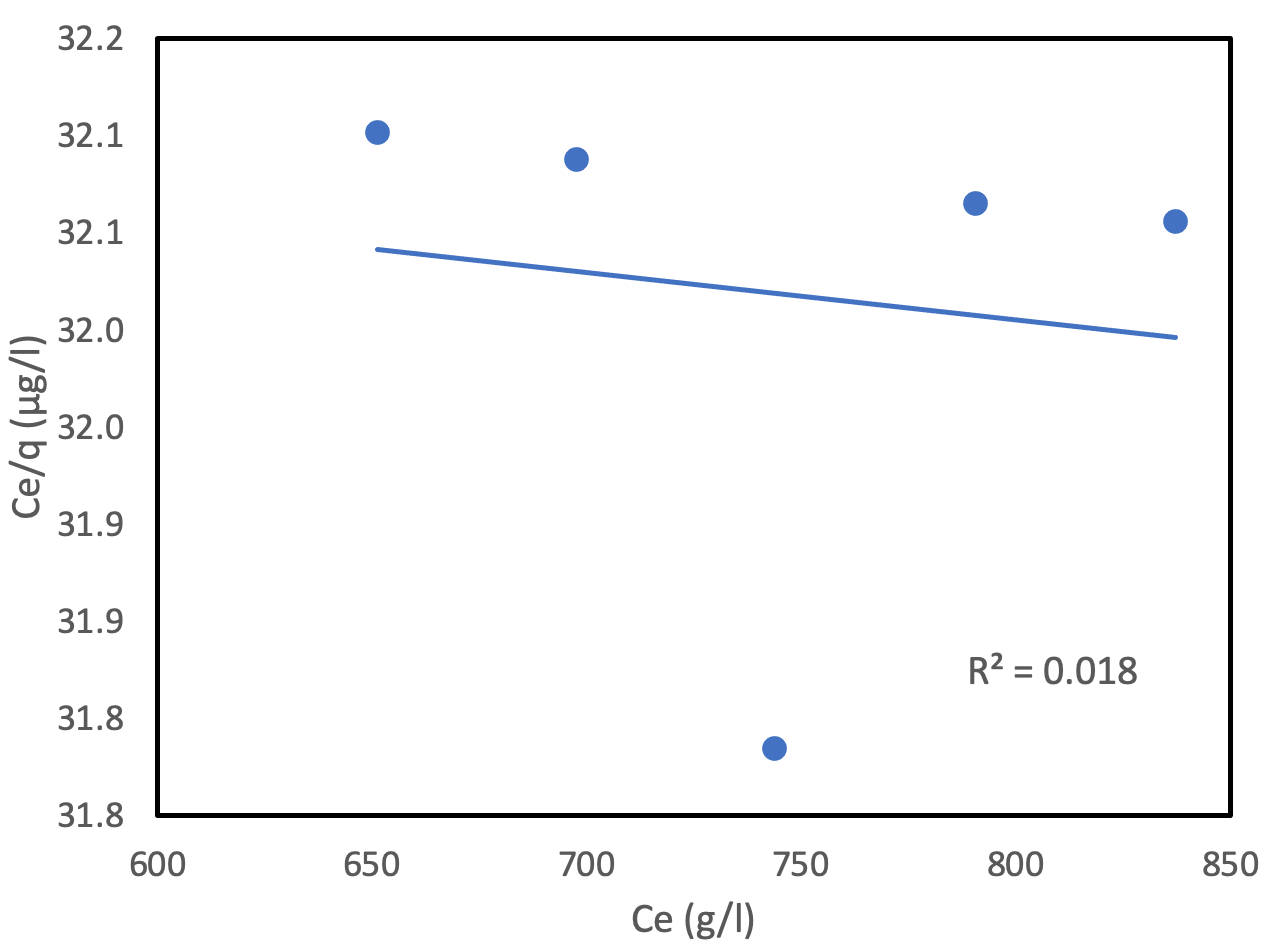


**Figure S6.** Plot of Langmuir isotherm model fitting to experimental data


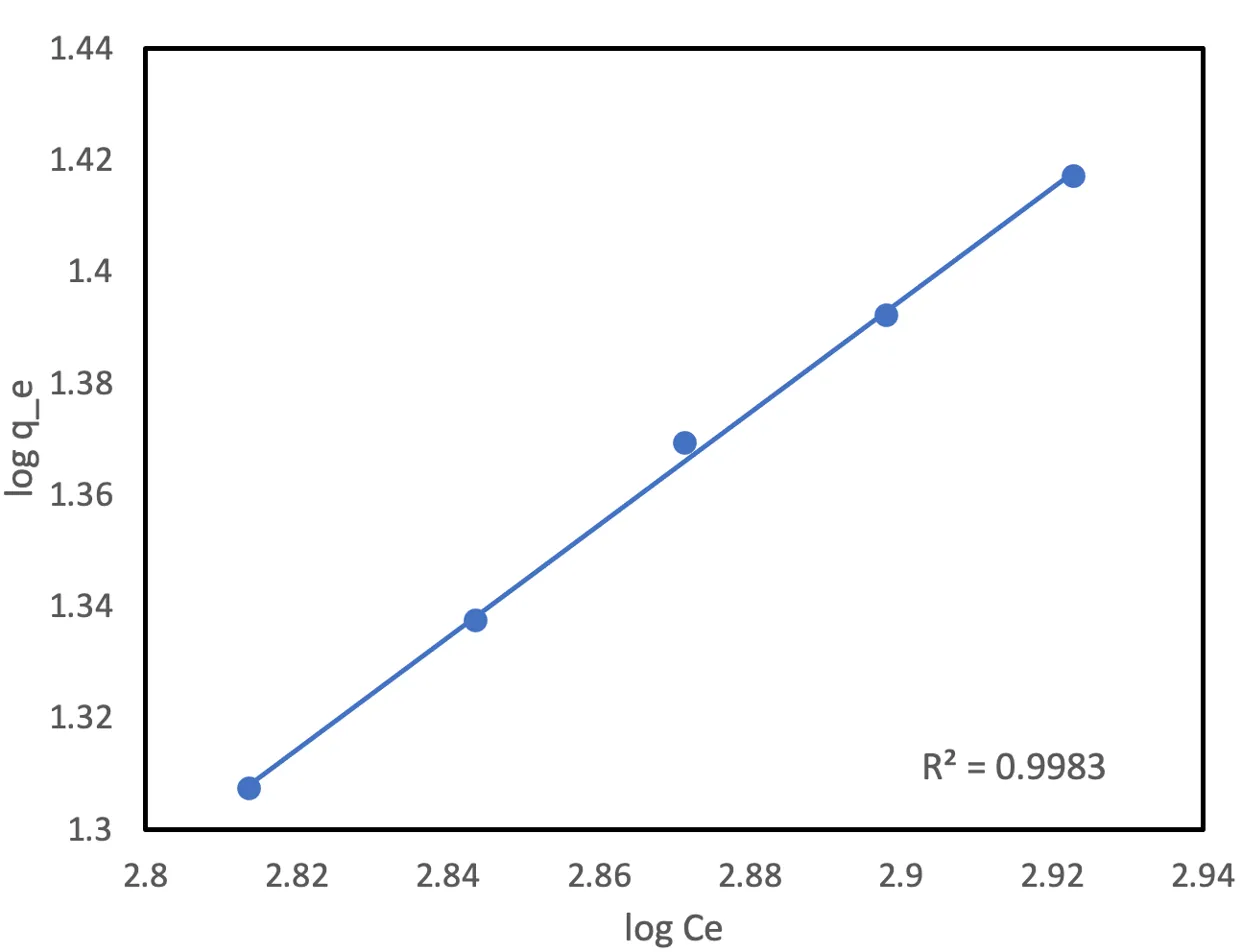


**Figure S7.** Plot of Freundlich isotherm model fitting to experimental data.


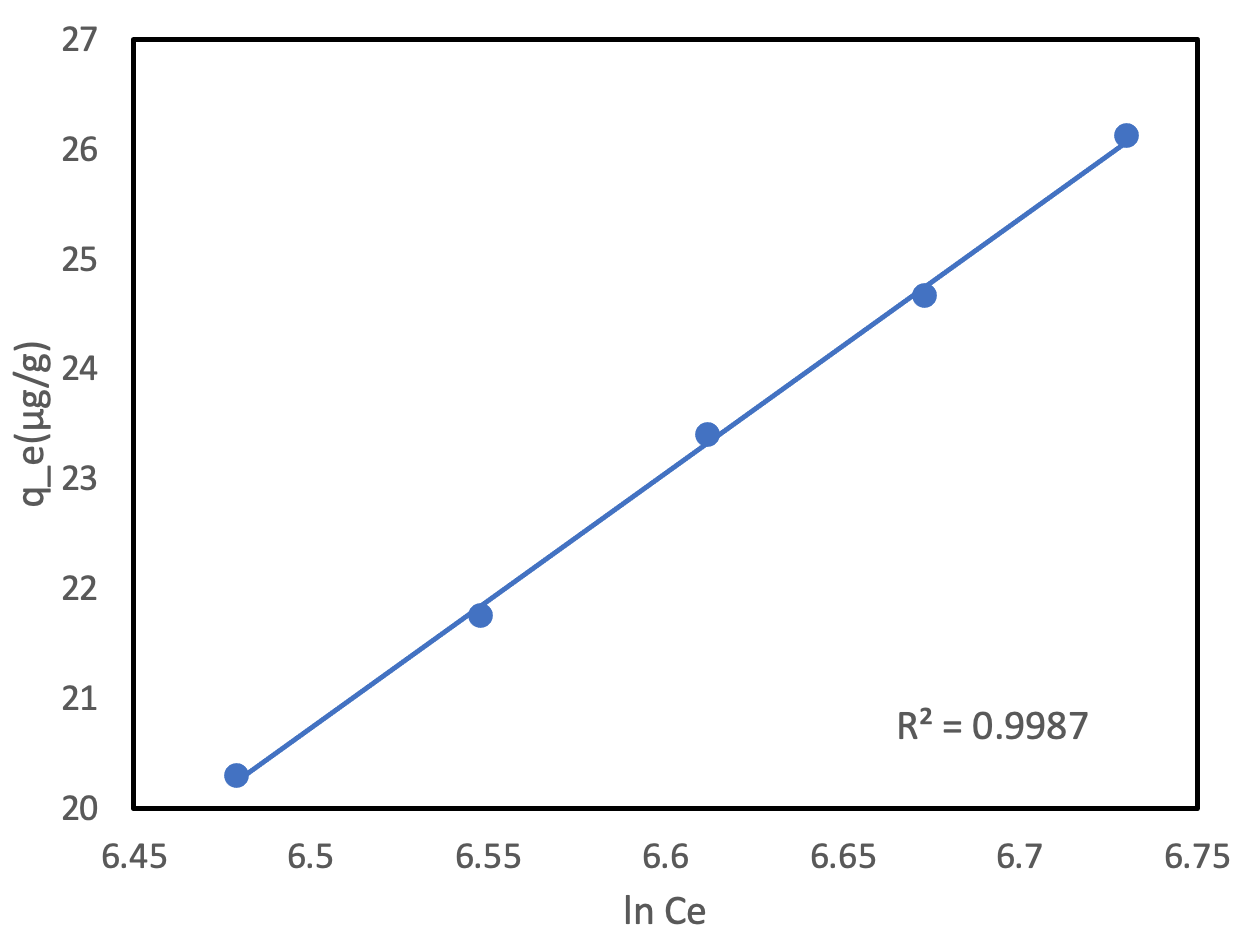


**Figure S8.** Plot of Temkin isotherm model fitting to experimental data.

The superior fit of both Freundlich and Temkin models compared to the Langmuir model indicates that DES adsorption onto PVDF membranes involves heterogeneous surface interactions and multilayer adsorption mechanisms, consistent with the complex morphology of PVDF membrane surfaces.
