# Supplementary material for: Adsorptive behavior of poly (vinylidene fluoride) membranes for the recovery of lignin-derived hydrophobic deep eutectic solvents
Source: Sci Rep. 2025 Sep 1;15:32051. doi: 10.1038/s41598-025-18164-x (PMC12402438; doi:10.1038/s41598-025-18164-x)
Supplement: Supplementary file 2 — Supplementary Material 2. [file 41598_2025_18164_MOESM2_ESM.docx]

ASSOCIATED CONTENT

Supporting Information. visual assessment of membrane polymer stability in deep eutectic solvent at different time intervals, FTIR spectroscopic analysis of PVDF membrane with and without polyethylene glycol pore-forming agent, DES concentration profiles over time at 40°C showing different initial concentrations, DES concentration profiles over time at room temperature (25°C) showing different initial concentrations, DES concentration profiles over time at 15°C showing different initial concentrations, Table of kinetic model parameters for DES adsorption, and isotherm model fittings including Langmuir, Freundlich, and Temkin models applied to experimental data.
